# Functional Connectivity of the Posteromedial Cortex

**DOI:** 10.1371/journal.pone.0013107

**Published:** 2010-09-30

**Authors:** Franco Cauda, Giuliano Geminiani, Federico D'Agata, Katiuscia Sacco, Sergio Duca, Andrew P. Bagshaw, Andrea E. Cavanna

**Affiliations:** 1 CCS fMRI, Koelliker Hospital, Turin, Italy; 2 Department of Psychology, University of Turin, Turin, Italy; 3 Department of Neuroscience, AOU S. Giovanni Battista, Turin, Italy; 4 School of Psychology, University of Birmingham, Birmingham, United Kingdom; 5 Department of Neuropsychiatry, University of Birmingham and Birmingham and Solihull Mental Health NHS Foundation Trust (BSMHFT), Birmingham, United Kingdom; 6 Institute of Neurology, University College London, London, United Kingdom; Indiana University, United States of America

## Abstract

As different areas within the PMC have different connectivity patterns with various cortical and subcortical regions, we hypothesized that distinct functional modules may be present within the PMC. Because the PMC appears to be the most active region during resting state, it has been postulated to play a fundamental role in the control of baseline brain functioning within the default mode network (DMN). Therefore one goal of this study was to explore which components of the PMC are specifically involved in the DMN. In a sample of seventeen healthy volunteers, we performed an unsupervised voxelwise ROI-based clustering based on resting state functional connectivity. Our results showed four clusters with different network connectivity. Each cluster showed positive and negative correlations with cortical regions involved in the DMN. Progressive shifts in PMC functional connectivity emerged from anterior to posterior and from dorsal to ventral ROIs. Ventral posterior portions of PMC were found to be part of a network implicated in the visuo-spatial guidance of movements, whereas dorsal anterior portions of PMC were interlinked with areas involved in attentional control. Ventral retrosplenial PMC selectively correlated with a network showing considerable overlap with the DMN, indicating that it makes essential contributions in self-referential processing, including autobiographical memory processing. Finally, ventral posterior PMC was shown to be functionally connected with a visual network.

The paper represents the first attempt to provide a systematic, unsupervised, voxelwise clustering of the human posteromedial cortex (PMC), using resting-state functional connectivity data. Moreover, a ROI-based parcellation was used to confirm the results.

## Introduction

The posteromedial cortex (PMC) is an architectonically discrete region comprising the retrosplenial areas (BA 29 and 30), the posterior cingulate areas (BA 23a,b,c), the mesial parietal area in the precuneus region (BA 7m), and BA 31, a transition area between BA 23c and BA 7m [1], [2]. Over the last few years, the PMC has received an increasing amount of attention because it has been identified as the most active brain region during a baseline state where healthy subjects are asked to lay in the scanner and ‘rest’ (i.e. the resting state). PET studies of healthy subjects in this resting condition have shown that the PMC consumes about 40% more glucose than the hemispheric mean [3]. Interestingly, the PMC and other brain regions, including the inferior parietal lobule (principally the angular gyrus), the superior frontal gyrus and the medial frontal gyrus, have been consistently found to be more active at rest than during non-self directed cognitive tasks. This observation has suggested the existence of a resting state in which the brain remains active in an organized manner, the so-called Default Mode Network (DMN). Recently, two research groups [4], [5] independently and in parallel proposed the analysis of connectivity in the resting human brain in term of two diametrically opposed brain networks, identified on the basis of both spontaneous correlations within each network and anticorrelations between networks; the authors have identified cortical foci for intrinsically defined anticorrelated networks, the task-positive network (TPN) and the task-negative network (TNN).

As well as its role in the DMN, the PMC has been implicated in a number of studies which have investigated the neural correlates of altered conscious states. The PMC shows selective deactivations during propofol-induced anesthesia [6], sleep [7], persistent vegetative state and coma [8], and stands out as the first brain region to show increased activity in patients regaining consciousness from drug-induced anesthesia and persistent vegetative state [2], [9], [10]. Alterations in PMC activity have also been detected in subjects experiencing pain [11], [12], and in patients with mild cognitive impairment, Alzheimer's disease [13], [14], [15] and other neuropsychiatric conditions, including epilepsy, schizophrenia, affective disorders and attention-deficit hyperactivity disorder (for a review [16]).

Anatomically, the cytoarchitectonic areas of the PMC are strongly inter-connected, and the PMC also has extensive external connections with higher-order association areas, namely the anterior cingulate, the mid-dorsolateral prefrontal, the lateral parietal cortices, the temporo-parieto-occipital area (TPO) [17], [18], as well as the dorsal-most sector of the thalamus [19]. Moreover, all PMC regions receive projections from the claustrum and the basal forebrain and project to the caudate, the basis pontis and the zona incerta [19]. Beyond these shared projections, the posterior cingulate areas are also interconnected with the parahippocampal regions. It has recently been suggested that different areas within the PMC have partly different anatomical connectivity patterns, suggesting that distinct functional modules may be present within the PMC [19]. One way of directly examining this issue in order to gain a more complete understanding of the functional sub-divisions of PMC is to investigate resting state functional connectivity (rsFC). In a recent study by Margulies et al. [20], resting state functional connectivity of the precuneus was compared between macaque monkeys and humans, with the results indicating comparable sub-divisions across species. Specifically, it was suggested that there are three functional sub-divisions of the PMC which are in reasonable agreement with previous anatomical tracer studies.

Over the past few years, intrinsic rsFC, as revealed by low-frequency spontaneous signal fluctuations in fMRI signal time-courses, has gained increasing attention in the neuroscience community. This approach has recently helped to clarify the functional connectivity of several brain regions, including the thalamus [21], insula [22], striatum [23], anterior cingulate cortex [24], red nucleus [25], cerebellum [26], and amygdala [27]. These findings have been shown to be consistent with meta-analyses of human functional imaging data [28], [29] and anatomical data from humans [30], [31], non-human primates [32] and rodents [33]. Moreover, multimodal imaging has demonstrated that functional connectivity in these intrinsic networks has well-defined electrophysiological signatures [34], [35], [36], [37].

Spontaneous resting state fluctuations of the blood oxygen level dependent (BOLD) functional magnetic resonance imaging (fMRI) signal reflect patterns of spatiotemporal synchrony, temporally coherent within anatomically and functionally related areas of the brain [5], [38], [39], [40]. Resting-state networks (RSNs) have been demonstrated in multiple systems within the cerebral cortex related to specific types of sensory, motor, and cognitive functions (for a review see [41]), and it has been suggested that up to ten RSNs are present in the human brain [38], [42].

In this study, we investigated the functional connectivity of the human posteromedial cortex during the resting state. Specifically, we employed two different approaches: a voxelwise unsupervised clustering technique and a ROI-based parcellation approach; these techniques let us determine whether BOLD fluctuations within the PMC correlate with changes in activity within other brain areas. Identifying specific patterns of resting connectivity within different anatomical regions of the PMC might suggest the existence of functional sub-units.

## Methods

### Ethics Statement

All subjects gave their informed written consent, in line with the Declaration of Helsinki, and the study was approved by the Ethics Committee of the Department of Psychology, University of Turin.

### Subjects

Seventeen right-handed healthy volunteers (9 female; mean ± standard deviation, 54±30.2 years old) participated in the study. None suffered from any neurological or psychiatric disorder, nor had a history of drug or alcohol abuse. None were on medications known to alter brain activity. All subjects were instructed simply to keep their eyes closed, think of nothing in particular, and not to fall asleep.

### Task and image acquisition

Subjects were instructed simply to keep their eyes closed, think of nothing in particular, and not to fall asleep. After the scanning session, participants were asked if they had fallen asleep during the scan with the aim to exclude subjects with positive or doubtful answers. No subjects were excluded from the study.

Images were gathered on a 1.5 Tesla INTERA™ scanner (Philips Medical Systems) with a SENSE high-field, high resolution (MRIDC) head coil optimized for functional imaging. Resting state functional T2^*^ weighted images were acquired using echoplanar (EPI) sequences, with a repetition time (TR) of 2000 ms, an echo time (TE) of 50 ms, and a 90° flip angle. The acquisition matrix was 64×64, with a 200 mm field of view (FoV). A total of 200 volumes were acquired, with each volume consisting of 19 axial slices, parallel to the anterior-posterior (AC-PC) commissure; slice thickness was 4.5 mm with a 0.5 mm gap. To reach a steady-state magnetization before acquiring the experimental data, two scans were added at the beginning of functional scanning, the data from these scans were discarded.

Within a single session for each participant, a set of three-dimensional high-resolution T_1_-weighted structural images was acquired, using a Fast Field Echo (FFE) sequence, with a 25 ms TR, an ultra-short TE and a 30° flip angle. The acquisition matrix was 256×256, the FoV was 256 mm. The set consisted of 160 contiguous sagittal images covering the whole brain. In-plane resolution was 1 mm×1 mm and slice thickness 1 mm (1×1×1 mm^3^ voxels).

### Data analysis

BOLD imaging data were analyzed using the Brain Voyager QX software (Brain Innovation, Maastricht, Holland). In order to reduce artifacts [43] and to improve statistical analysis functional images were preprocessed as follows: (1) Slice scan time correction was performed using a sinc interpolation algorithm. (2) 3D motion correction: all volumes were aligned spatially to the first volume by rigid body transformations, using a trilinear interpolation algorithm; the roto-traslation information were saved for subsequent elaborations. (3) Spatial smoothing was performed using a Gaussian kernel of 8 mm FWHM. (4) Temporal filters were used to reduce cardiac as well as respiratory noise: linear trend removal and band pass filter of 0.01–0.1 Hz was used as several previous studies [39], [44] have found the range of frequency [0.1-0.01 Hz] to have the greatest power in revealing the underlying connectivity [13], [44], [45], [46], [47].

After pre-processing, a series of steps were followed in order to allow for precise anatomical location of brain activity and to facilitate inter-subject averaging.

First, each subject's slice-based functional scan was co-registered on his or her 3D high-resolution structural scan. Second, the 3D structural data set of each subject was skull-stripped and transformed into Talairach space: the cerebrum was translated and rotated into the anterior-posterior commissure plane and then the borders of the cerebrum were identified. Third, the volume time course of each subject was created in the subject-specific anatomic space. The Talairach transformation of the morphologic images was performed in two steps. The first step consisted of rotating the 3D data set of each subject to align it with the stereotactic axes. In the second step, the extreme points of the cerebrum were specified. These points were then used to scale the 3D data sets to the dimensions of the standard brain of the Talairach and Tournoux atlas using a piecewise affine and continuous transformation for each of the 12 defined subvolumes.

Intersubject coregistraton was performed at the cortex-level using a cortex-based high-resolution intersubject alignment (see supplementary materials for further details). Only for group statistics the individual maps were projected onto the normalized volumetric image using volumetric anatomy.

### ROI selection

**Figure 1:**
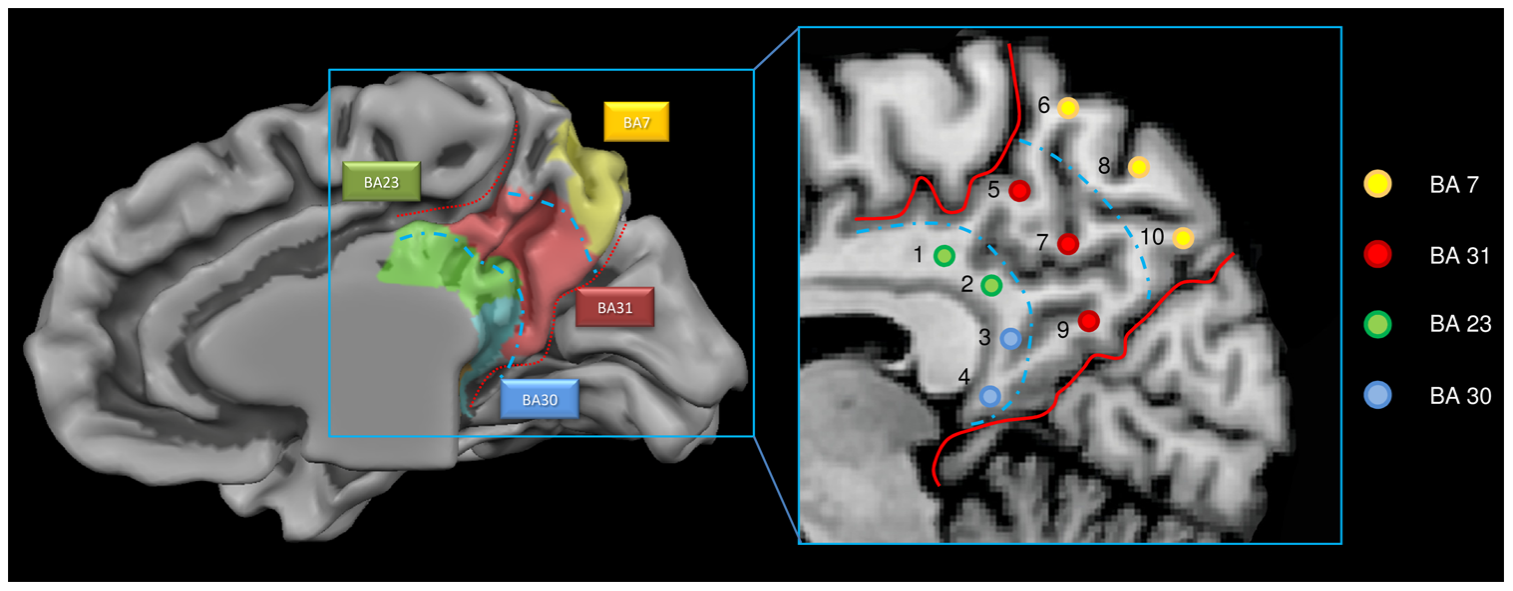
ROI used as seed regions. ROI used as seed regions for rsFC analyses and Brodmann subdivision of the area object of our study.

The goal of the present study was to provide a systematic map of functional connectivity of the PMC. To examine the functional connectivity of all the PMC parenchyma, we created a template with all subjects' anatomical images and we drew ten seed ROIs over the template's 3D renderized PMC surface in an equispaced fashion taking into account previous anatomical studies [19] each seed measures 125 mm^3^. See Fig 1 and Supporting file S2. More specifically, on the previously created template, we traced a curve within the posterior cingulate cortex, using the callosal curve as a reference, and over this line we placed 4 equispaced ROIs. Subsequently, over the remaining PMC cortex, we drew 3 lines in the rostral-caudal or dorso-ventral direction, and on each of the 3 lines we drew 2 ROIs, for a total of 10 equispaced ROIs.

To restricted each ROI to gray matter, we segmented each subject's anatomical image and we created gray matter (GM) probabilistic maps using the Brainvoyager VOI analysis Tool. Each VOI was placed in areas of very high subject-specific GM overlap ( = >70%), carefully delineated to avoid the multicollinearity caused by excessive proximity between ROIs. To circumvent the same problem, the maximum number of ten bilateral ROIs was chosen following the empirical suggestions of Zhang and Snyder [21]. All ROIs are listed in supporting file S2.

To test whether bilateral ROIs were leading to uncorrect estimation of high lateralized networks, we first computed the analyses with unilateral ROIs; as the results of right and left ROIs were highly similar, we proceeded using bilateral ROIs (see Supporting file S2).

Additional analyses were conducted using alternative ROIs, i.e. ROIs which were moved from the original location in the dorsal, rostral and caudal directions (3 mm in each direction), and reduced (27 mm^3^) and increased (512 mm^3^) in dimensions. We found high similarity between the resulting maps and those obtained using the original ROIs: probabilistic maps showed high overlapping between the original and the alternative connectivity maps (see Supporting file S2).

### Functional connectivity analysis

FC maps were computed according to Margulies et al. [24]. BOLD time courses were extracted from each ROI by averaging over voxels within each region. Several nuisance covariates were included in the analyses to reduce the effects of physiological processes such as fluctuations related to cardiac and respiratory cycles [48], [49] or to motion. We included 9 additional covariates that modeled nuisance signals from White Matter, Cerebro-Spinal Fluid, Global Signal [50], [51], as well as 6 motion parameters (3 rotations and 3 translations). All seed-based predictors were z-normalized, and orthogonalized to each other, to ensure that the time series for each ROI reflected its unique variance.

To test whether orthogonalization was leading to underestimation of functional connectivity, analyses were repeated with each insular subdivision in a separate regression model. Results were highly similar to those found with orthogonalization (see Supporting file S2); therefore, only the orthogonalized results are presented here.

Temporal autocorrelation correction (Pre withening) [52] was used. Seed ROI-driven FC maps were computed on a voxel-wise basis for each previously selected region. The individual participant multiple regression analysis was carried out using the general linear model (GLM) [53] and resulted in a t-based map (SPM-t) corrected for multiple comparisons at the cluster level using a Monte Carlo simulation ([54], [55], see supporting file S1, p<0.05), leading to a cluster threshold K>16 voxels in the native resolution).

### Group statistical map

Random effect group-level analyses, controlling for age and gender effects, were conducted using the ANCOVA analysis tool implemented in BrainVoyager QX. Corrections for multiple comparisons were performed at the cluster level using a Monte Carlo simulation ([54], [55], see supporting online materials, p<0.05), leading to a cluster threshold K>19 voxels in the native resolution); the resulting maps were then projected on a partially inflated (22%) 3D representation of a template using the BrainVoyager QX cortical tool.

To evaluate the spatial consistency of functional connectivity patterns across subjects we computed spatial probabilistic maps. Probability maps were calculated separately for each ROI-generated network. The probability map describes the relative frequency (expressed in percentage) with which the same network is represented over different brain areas.

To help the result visualization and analysis e created a Maltab® script, see see Supporting file S1 for a detailed description.

### Voxelwise parcellation

We applied fuzzy clustering on each unilateral unsmoothed Posteromedial part of parenchyma to achieve a voxelwise segregation of the underlying PMC networks. PMC gray matter meshes were segmented from each subject morphological image and coregistered using the BrainVoyager QX high-resolution intersubject cortex alignment (see supplementary methods). Voxels belonging to this region were submitted to a voxelwise unsupervised fuzzy clustering technique as implemented in the BrainVoyager QX Fuzzy clustering plugin [56]. Fuzzy clustering partitions a subset of *n* voxels in *c* “clusters” of activation [56], [57]. The *z*-standardized signal time courses of all voxels are simultaneously considered, compared, and assigned to representative cluster time courses (cluster centroids). This data-driven method thus decomposes the original fMRI time series into a predefined number of spatiotemporal modes, which include a spatial map and an associated cluster centroid time course. The extent to which a voxel belongs to a cluster is defined by the similarity (as measured, e.g., by correlation) of its time course to the cluster centroid. In this method, “fuzziness” relates to the fact that a voxel is generally not uniquely assigned to one cluster, but instead, the similarity of the voxel time course to each cluster centroid is determined. This is expressed by the “membership” *u_cn_* of voxel *n* to cluster *c*. Cluster time course and membership functions are updated in an iterative procedure [58] that terminates when successive iterations do not further change memberships and cluster centers significantly as determined via classical cluster algorithm distance measures. We ensured an optimal implementation of the fuzzy clustering by performing an unsupervised search for the optimal number of clusters (see Supporting file S1 for a detailed description) leading to a number of four clusters. As suggested in literature [59], [60], [61], [62], we set the parameter “m” controlling the degree of fuzziness to a value within the range of values commonly used in Fuzzy C-Means algorithms using fMRI datasets (0.4), which allows some voxels to be classified in more than one cluster. We applied principal component analyses to the datasets to reduce dimensionality while capturing at least 90% of the total variance/covariance. Single subject maps were grouped using the SogIca method (see Supporting file S1), group-level results were visualized using probabilistic maps. The resulting probabilistic maps were reported in the interval [10–100%] and superimposed on the inflated representation of a template brain (average brain).

### Reliability test

To evaluate the spatial consistency of functional connectivity patterns across subjects we computed the Split half reliability index: we calculated the reliability coefficient with the Spearman Brown [63], [64] formula, 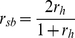 where the term *r_h_*, in our case is the spatial similarity of the maps obtained by two random selected, equally numerous subgroups. The *r_h_* term is a measure of the intersection of two fuzzy sets, the Sørensen index [65], defined as: 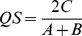, where A and B are the elements in sample A and B, respectively, and C is the number of elements shared by the two samples (this is equivalent to Dice metric).

## Results

After inspecting the motion-correction parameters we excluded one subject because of movements exceeding 1 mm translation/1° rotation. The revised demographics were as follows: sixteen right-handed healthy volunteers (8 female; mean age 53; age range 23–75 years).

### Voxelwise parcellation

**Figure 2:**
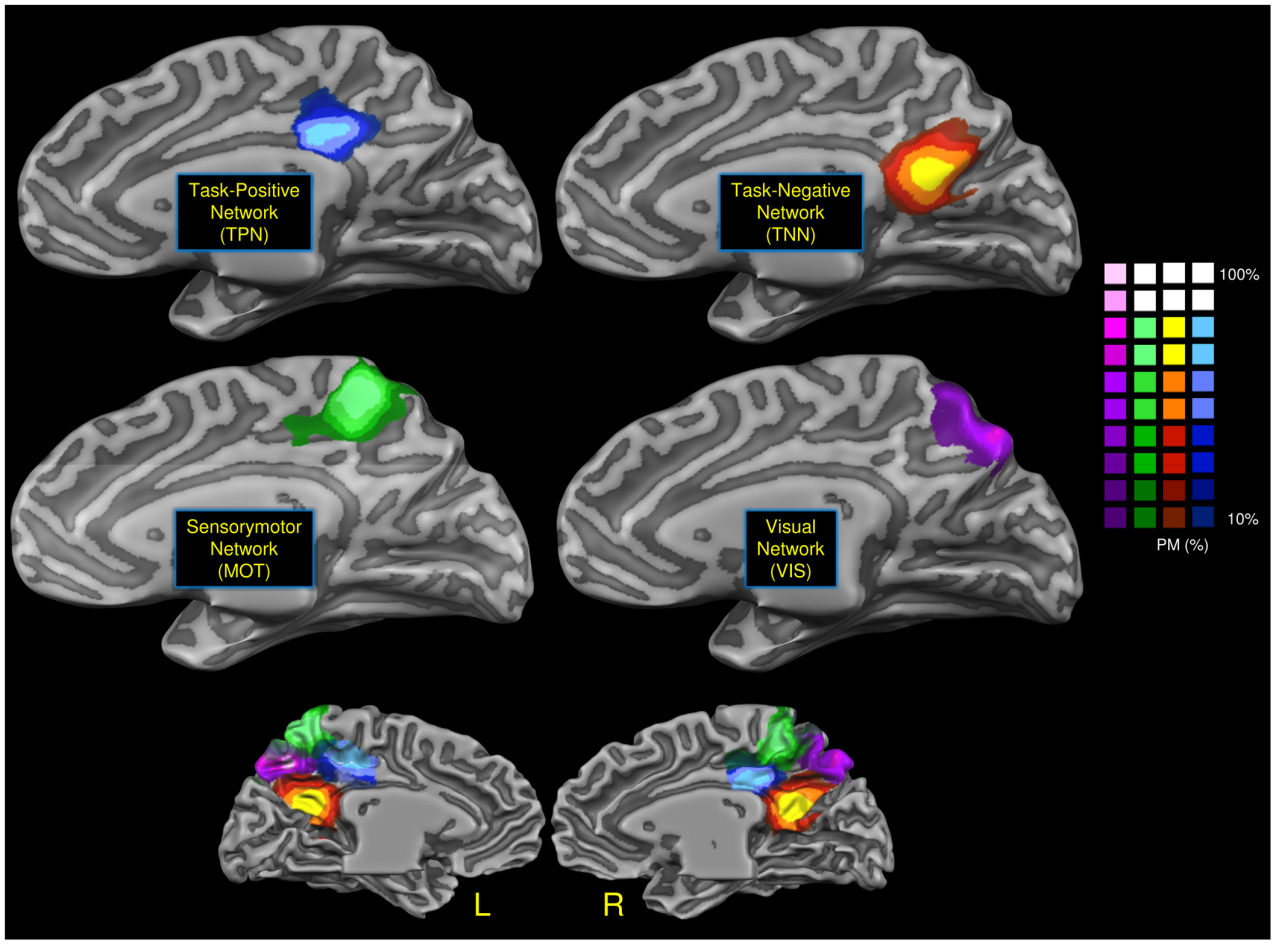
Voxelwise clustering. Connectivity-based parcellation of human posteromedial cortex. Probabilistic maps for functional connectivity-defined clusters. The color scheme represents the probability of overlapping brains in each voxel across the 16 subjects. Maps are projected on a inflated 3D brain surface with the BrainVoyager QX surface tool.

Fig 2 shows the four clusters obtained through the voxelwise clustering algorithm: each cluster evidenced a different network connectivity. In the dorsal anterior part of PMC prevalently in the territory of BA 23 we recognize a pattern that show a functional connectivity with the TPN. Posteriorly and dorsally to this cluster, in the territory of BA 7 and 31 we evidenced a cluster with a strong sensorimotor connectivity (MOT). In the lowermost posterior part of BA 7 we found an area characterized by visual connectivity (VIS). Finally in the ventral retrosplenial area, in the territory of BA 30, 31 and partially 23, we recognize a cluster with correlations with the TNN. Our results are fully in agreement with the results of Margulies et al. [20]: see their figure S6b and our in the supporting file S2.

### ROI-based parcellation

#### Interconnections among PMC components

**Figure 3:**
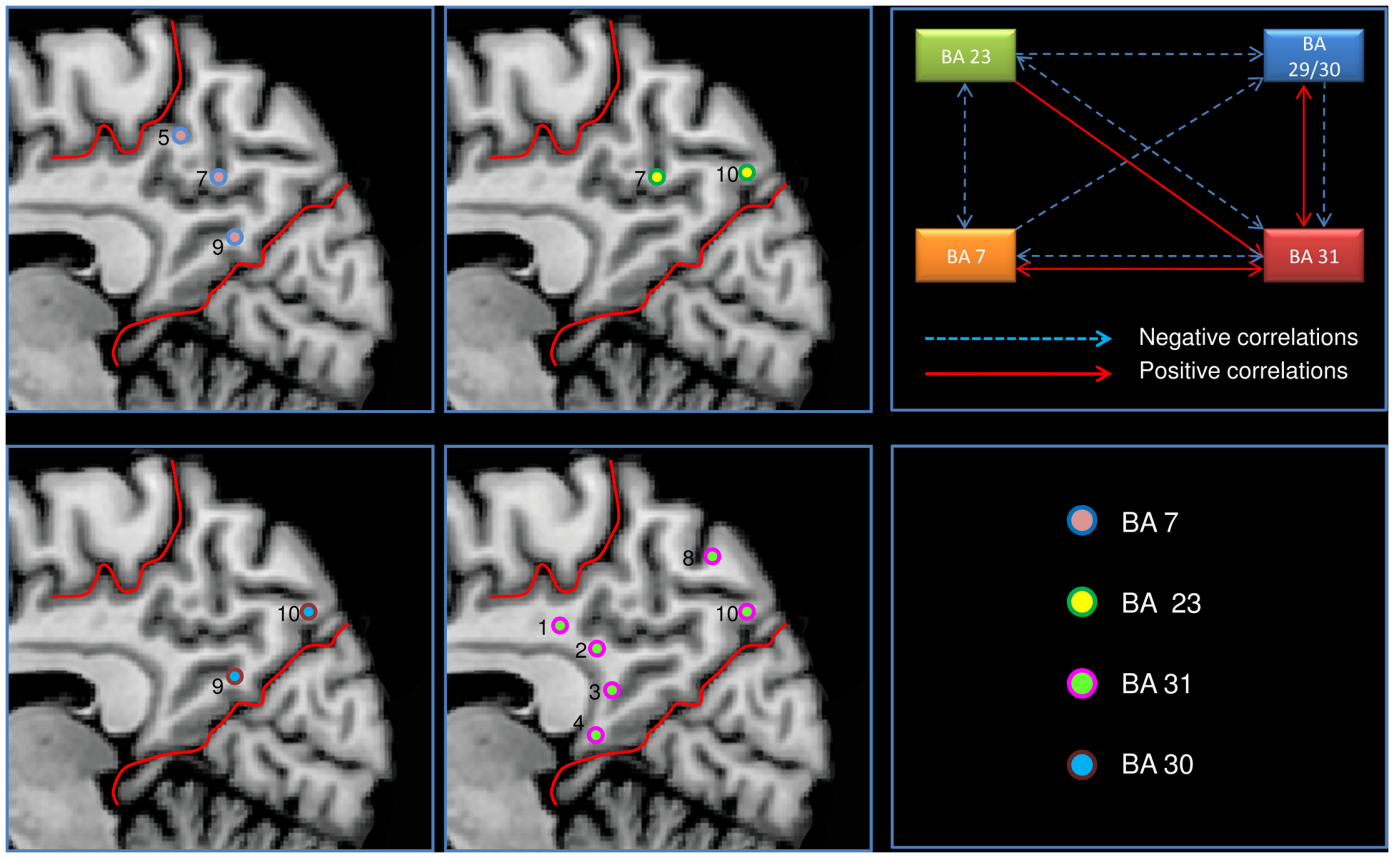
Correlations of each ROI with Brodmann's area intra PMC. a) left and middle panels: Correlations of each ROI with Brodmann's area intra PMC. b) right upper panel: Correlations intra PMC (BA  =  Brodmann's area).

Fig 1 shows the anatomical locations of the ten ROIs (details and a more analytical description is given in the Supporting file S2). We found a high level of local connectivity in each of the ten seed-generated ROI maps: each ROI had a wide area of local connections within PMC. To avoid this result may be influenced by the presence of spatial smoothing we repeated the analysis without using any spatial smoothing. ROIs 2, 4, 7 and 8 had a particularly high local connectivity while 3, 6 and 10 had much less local connectivity (see Supporting file S2). The posterior part of PMC (ROI 3, 4, 9, 10) is correlated with BA 30, whereas the more rostral region of PMC is correlated with BA 23; finally only BA 31 is correlated with BA 7m. Fig 3 shows the reciprocal correlations among the different areas within the PMC; the complete pattern of reciprocal positive and negative correlations was only found between BA 31 and BA 7m.

#### Cortical connectivity

**Figure 4:**
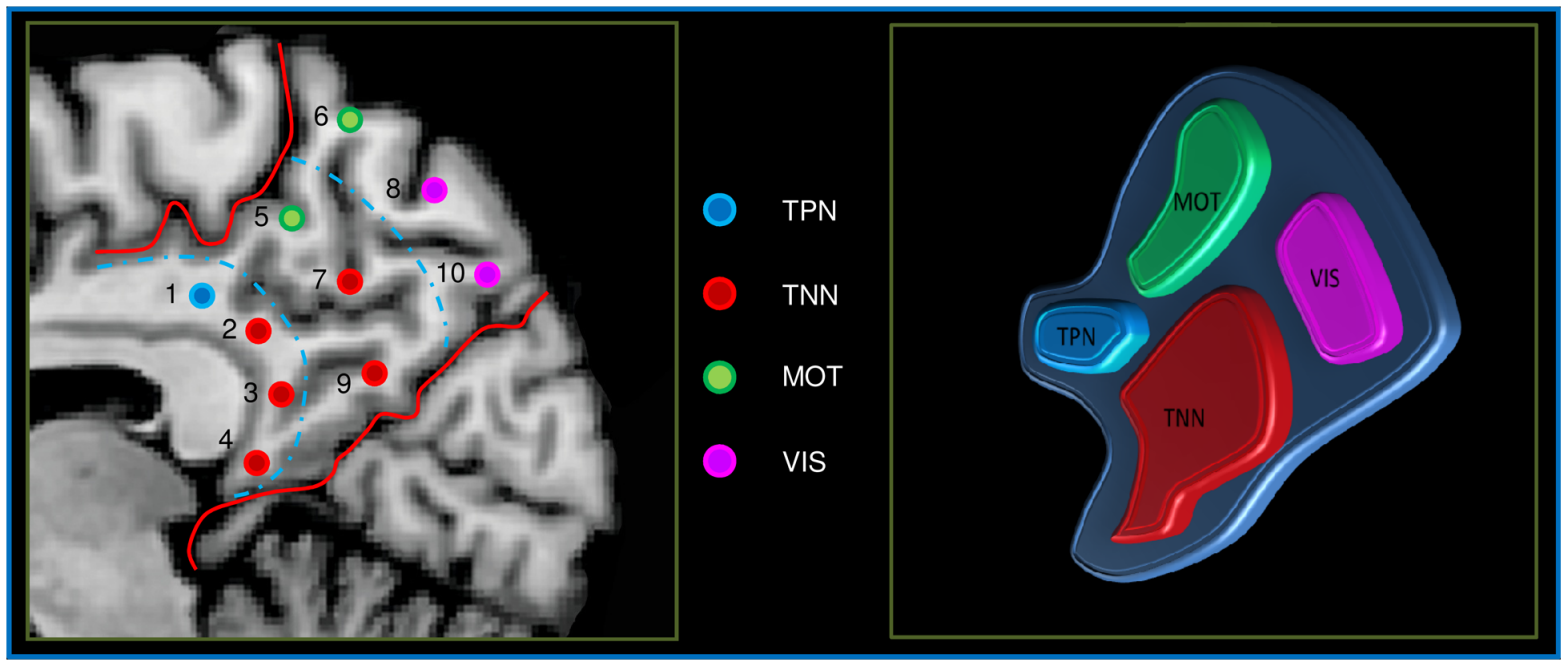
ROI-based parcellation. ROI-based parcellation of human posteromedial cortex. Spatial distribution of the four network found in the PMC.

**Figure 5:**
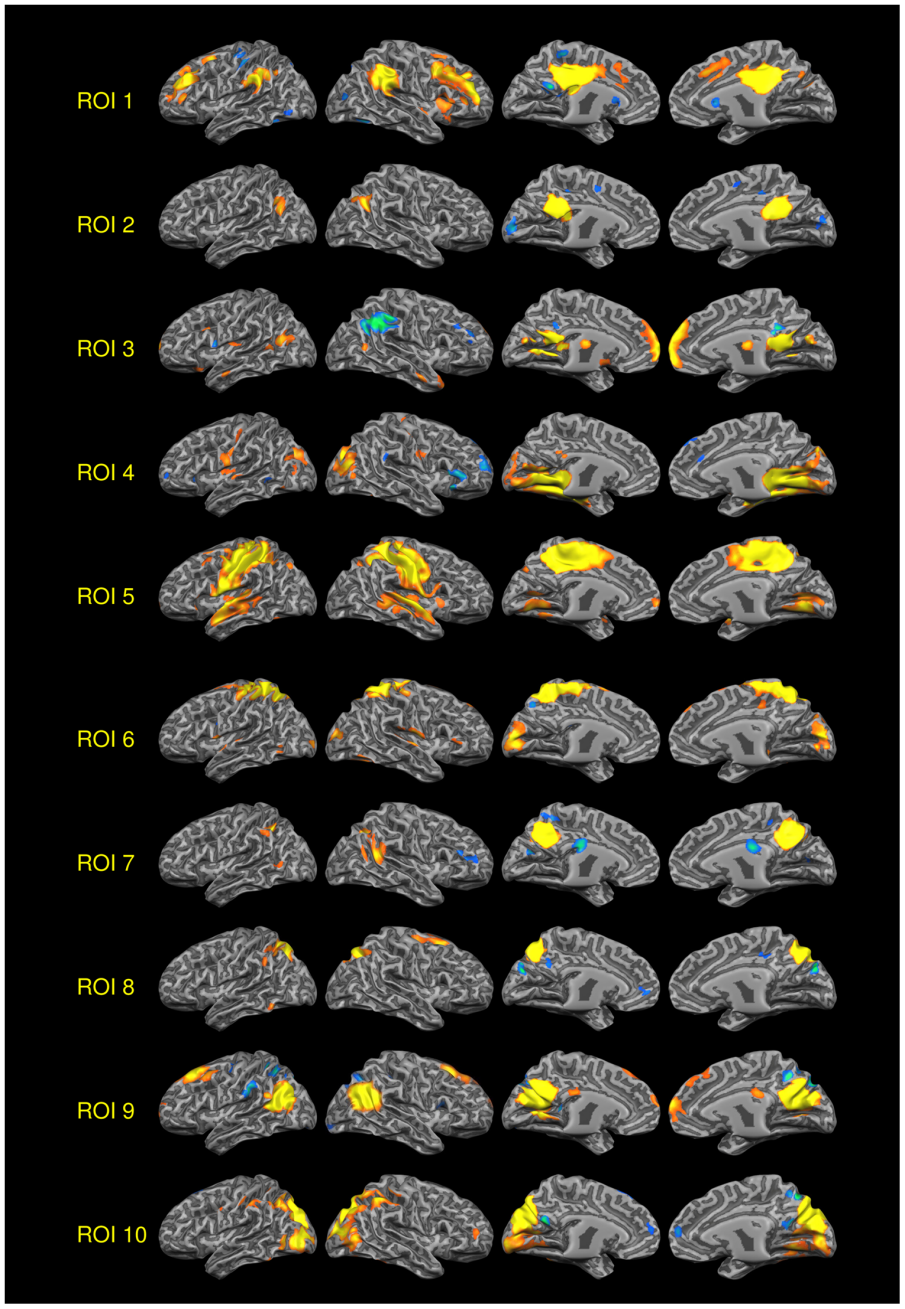
rsFC of all the 10 PMC ROIs. VOI 1–10 rsFC Positive and negative correlations (One sample t-test, corrections for multiple comparisons performed at the cluster level using a Monte Carlo simulation, see supporting online materials) (p<0.05), leading to a cluster threshold K>19 voxels in the native resolution);. Colors from red to yellow indicate positively correlated voxels. Colors from blue to green indicates negatively correlated voxels). Maps projected on a 3D average brain with the Brainvoyager QX surface tool.

Fig 4 shows the pattern of connectivity of each Seed ROI. Coherently to the voxelwise clustering, we evidenced four different patterns of functional connectivity: ROIs 5 and 6 placed in the most dorsal posterior part of the PMC placed in the dorsal territory of BA 7 and 31 are involved in sensorimotor activity (MOT). ROIs 2, 3, 4, 7 and 9, placed in the ventral retrosplenial cortex correspondent to BA 30 and 31 and part of BA 23 are prevalently involved in the TNN, a network often referred as DMN. ROI 1, placed in the anterior part of BA 23 is involved in the TPP, a network involved in attentional tasks. Finally the ROIs 8 and 10 placed in the most ventral posterior part of BA 7 are involved in the visual network (VIS). Fig 5 shows the rsFC of all 10 ROIs involved in this analysis (see Supporting file S2 for the conjunction analysis of all the ROIs and for a discussion of the ROIs lateralization).

**Figure 6:**
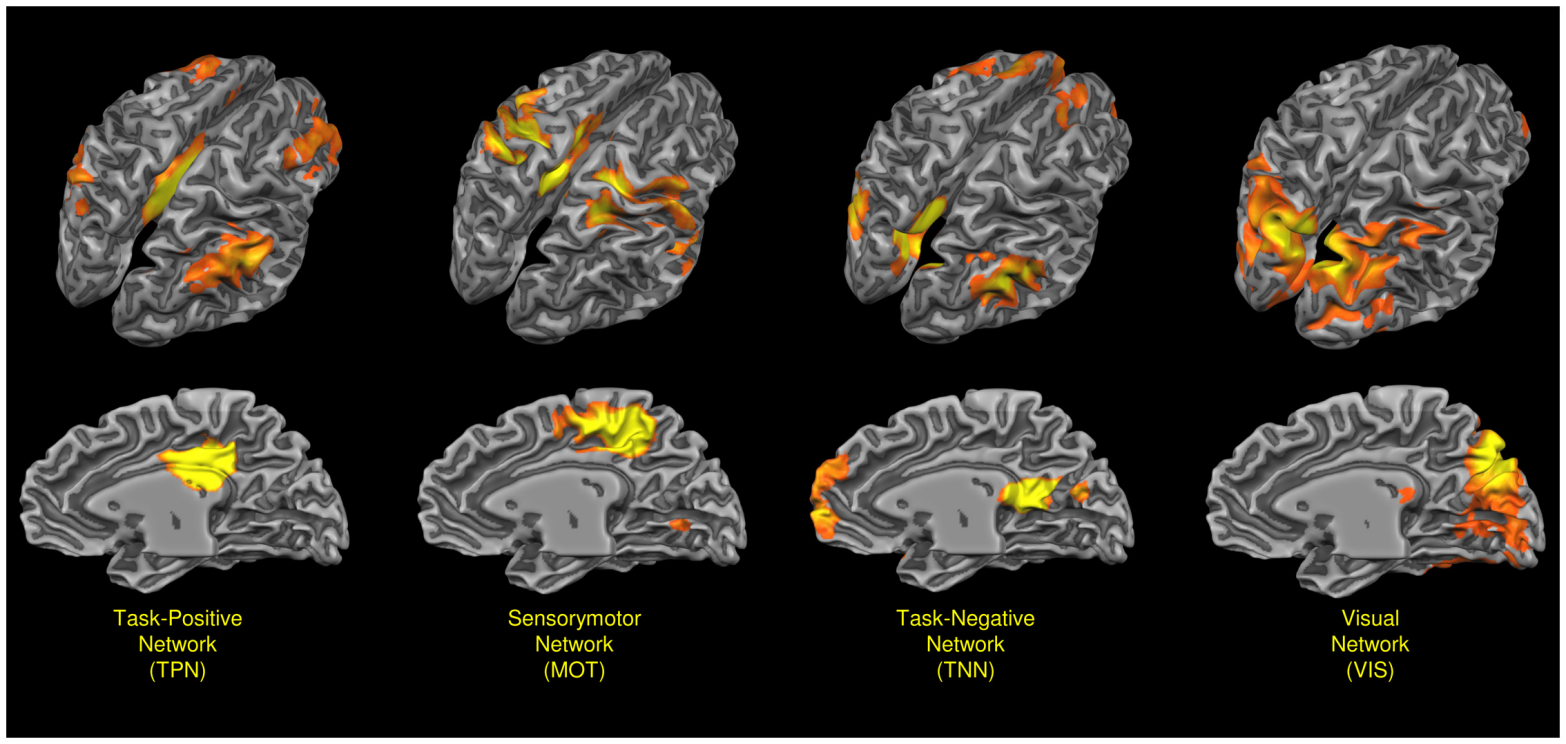
Prevalent networks found in the PMC. Probabilistic maps of all the four prevalent networks found in the PMC. Probability mapped from 35 to 70%. Maps projected on a 3D average brain with the Brainvoyager QX surface tool. TNN: Task-Negative Network, TPN: Task-Positive Network, MOT: Sensorimotor Network, VIS: Visual Network.

All the four networks found in this region are visualized in Fig 6. Though some ROIs express a clear correlation with the four previously described patterns, the remaining ROIs present mixed patterns of connectivity that are a blend of rsFC patterns of the surrounding areas.

#### Subcortical connectivity

Generally, the activation associated with a positive subcortical and cerebellar correlation had a greater volume than negative associated ones, further a greater number of areas did not show any negative correlation (no negative  =  ROI 2, 3, 6, 8; no positive  =  ROI 2, 7); ROI 2 is the only ROI that did not correlate with subcortical areas, ROI 9 had a quite similar pattern but had a very small positive correlated area in the left Culmen of cerebellum. (see Supporting file S2).

#### Thalamus

ROIs 1 and 3 were characterized by slow oscillations positively correlated with Thalamus' MD, VL, pulvinar and VPL nucleus, with a peculiar lateralization pattern (ROI 3 correlated with the left side only and ROI 1 with the right side only). ROI 7 negatively correlated with Thalamus VL right nucleus; ROI 5 and ROI 9 negatively correlated with Pulvinar, right side and left side respectively (see Supporting file S2).

#### Subcortical Limbic regions

ROI 4 had bilateral correlations with Amygdalae (more right than left), and Hippocampi (more left than right). ROI 6 also correlated with Amygdala, but only on the right side and the activation was significantly less extended. ROI 1 showed little negative correlation with bilateral Hippocampi (see Supporting file S2).

#### Basal Ganglia

Correlations with Basal Ganglia were negative, with the exception of ROI 3 which showed a positive correlation with the left caudate. ROI 1, 4, 5, 7 and 10 negatively correlated with basal ganglia activity (ROI 1: little dimension left Caudate, ROI 4: Bilateral Caudate with a strong right lateralization, ROI 5: Bilateral Caudate with a right predominance, ROI 7: right Putamen, ROI 10: right Putamen-Globus Pallidus). ROI 3 positively correlated with left ventral striatum (nucleus Accumbens). Finally, there was an extended connectivity pattern with the Claustrum ROI 1, 3, 5: positive correlations; ROI 7 and 9: negative correlations) (see Supporting file S2).

#### Cerebellum

The nearly total correlations with the Cerebellum were positive with the exception of ROI 5, which showed a negative correlation with the left Uvula and Pyramis (the activation is really very proximate to the medial line). ROI 3, 4 and 5 correlated with Culmen Bilaterally (ROI 5: left predominance), ROI 6 with Bilateral Culmen, Declive, Tuber, Pyramid, Nodule, Inferior Semi-Lunar Lobule, Cerebellar Tonsil and Vermis, ROI 8 with Bilateral (right predominance) Culmen, Uvula, Pyramis, Cerebellar Tonsil, ROI 9 with the left Culmen (small cluster), ROI 10 with Bilateral Culmen, Declive, and Tuber.

The anterior/medial cerebellum seemed to be positively correlated more with anterior/inferior PMC ROIs while the posterior/lateral cerebellum seemed to be positively more correlated with posterior PMC (see Supporting file S2).

BA 23 and BA 29/30 have correlations with the DM thalamus (connecting with prefrontal cortex); in particular, BA 29/30 correlates with cortical-subcortical loop of central network of motivation and reward of the left side (mesial prefrontal cortex, ventral striatum-accumbens, DM thalamus) and with amygdala. BA 31 has negative correlations with right motor subcortical loop (VL thalamus and putamen); moreover, BA 31 is negatively connected with the pulvinar and caudate. Both BA 23 and 29/30 have positive correlation with the Claustrum (right and left respectively), whereas BA 31 has both positive and negative correlations with the Claustrum. BA 7 has correlations with the cerebellum and with right putamen-pallidum; moreover, BA 7 has a positive correlation with the right amygdala. (see Supporting file S2).

### Reliability index

The split-half test performed with the Spearman Brown method between each ROI in the two split groups show that our results (Tab S5) have a good-to-high reliability (RSB>0.60, mean 0.67).

## Discussion

This paper provides a systematic mapping of the resting-state functional connectivity of PMC using a true voxelwise method. We used a ROI-based parcellation as previously employed by Margulies et al. [20] to confirm our results. Previous efforts to provide a comprehensive examination of functional connectivity in PMC using task-based approaches have required the synthesis of findings across multiple studies via meta-analysis [28], [29]. We hypothesized that the application of correlational analyses to resting-state fMRI data in a single study can enable the characterization of task-independent patterns of functional connectivity with more subtle regional differentiations. Therefore, we conducted an unbiased study to examine both cortical and subcortical functional correlations of all cytoarchitectonic areas within the PMC, using (i) a fuzzy clustering approach, clusterizing in a voxelwise fashion all the PMC parenchyma and (ii) a ROI-based parcellation approach involving the creation of 10 equispaced ROIs along four parallel curves aligned with the corpus callosum in each hemisphere.

### Intrinsic and shared correlations among PMC components

Our findings indicate that distinct cytoarchitectonic areas in the PMC are functionally correlated with each other, and the local intercorrelations are stronger between immediately adjacent areas than areas further apart. Interestingly, these results reflect previously obtained connectivity data from the macaque brain, which is the closest approximation to the human brain in conventional anatomical tracing experiments [18], [19]. For instance, almost all PMC areas (BA 7m, B23 and BA 29, located within the precuneus, posterior cingulate cortex and retrosplenial cortex) showed both positive and negative correlations with BA 31, which is an architectonically transitional area between BA7m and BA23. Moreover, according to voxel distance calculations, ROI located within the same cytoarchitectonic boundaries tended to be more strongly correlated.

Our results suggest a progressive shift in PMC functional connectivity from anterior to posterior and from dorsal to ventral ROIs. That is, dorsal posterior portions of PMC (i.e. the dorsal posterior part of BA 7 and 31 - ROIs 5 and 6) were shown to be part of a fronto-parietal network implicated in the visuo-spatial guidance of movements, whereas dorsal anterior portions of PMC (dorsal anterior part of BA 23 - ROI 1) were interlinked with areas involved in attentional control. The ventral anterior PMC (BA 30 and the ventral part of BAs 23 and 31 - ROIs 2–4, 7, 9) selectively correlated with a network showing considerable overlap with the DMN (TNN), the exact function of which has not been elucidated, although a central role for self processing and self awareness has been suggested [2]. Finally, the ventral posterior PMC (ventral part of BA 7 - ROIs 8 and 10) was shown to be functionally connected with a visual network.

The convergence of anatomical interconnectivity and functional intercorrelation maps provides strong evidence for the identification of a functional unit in baseline resting state activity [66]. This hypothesis is consistent with the considerable overlap between the shared connectivity pattern of PMC areas and the TNN (DMN). It has been shown that in the primate brain all PMC components are interconnected with the anterior cingulate gyrus, the mid-dorsolateral prefrontal cortex (area 46 and, to lesser extent, area 9), the lateral parietal cortex, and the TPO [19]. In addition to these regions, we found that the PMC has extensive functional correlations with other bilateral cortical areas within the frontal lobe, such as the VMPFC and the motor/supplementary motor cortex. These minor differences compared to primate data could be merely due to methodological differences between studies. Alternatively, they can be related to inter-species differences, with a more prominent role for fronto-parietal connections in Homo Sapiens. Moreover, functional connectivity studies disclose not only direct (anatomical) connections, but additional connectivity patterns which are mediated by other brain structures.

Beyond this shared connectivity pattern, our results showed progressive shifts in PMC functional connectivity from anterior/rostral to posterior/caudal and from dorsal to ventral ROIs. These findings suggest functional heterogeneity within the PMC (cfr. [20]). For example, although ROI 5 and 6 belong to the same sensorimotor cluster, the superior part of the precuneus (BA 7m) is characterized by its selective correlation pattern with the lateral parietal cortex, intraparietal sulcus (BA 5,7), inferior parietal lobule (BA 40), temporal neocortex (BA 20,21,22,37,38), visual cortex (BA 17,18,19), cerebellum and amygdala. The connectivity study by Parvizi et al. [19] showed a similar pattern of connections with frontal and cingulate structures involved in execution or planning of actions. In addition, the more rostral portion of BA 7m shows a broad pattern of connections with the motor and premotor cortex, the cerebellum, the visual system, and the insula. These findings are consistent with converging evidence suggesting that the anterior portion of the precuneus subserves visuo-spatial coordination skills required for reaching and grasping behaviors [2]. Specifically, the integration of mental imagery with sensorimotor and cerebellar information is thought to provide visual guidance to hand movements in conjunction with the superior parietal lobule [2], [67], [68], [69], [70]. Moreover, the results of our functional correlation analysis support the existence of a topographically-specific organization in the reciprocal parieto-frontal connections first proposed by Cavada and Goldman-Rakic [68] and subsequently confirmed by Leichnetz [18], such that the precuneus has important cortico-cortical connections with the rostral-most dorsal premotor cortex (BA 6, frontal eye field). Of note, experimental studies of electrical stimulation of BA 7m have resulted in saccade-like eye movements, thus suggesting the existence of another oculomotor center within the precuneus, a “medial parietal eye field” [71]. Cavanna and Trimble [2] have proposed that visuo-spatial information processing and spatially guided behavioural tasks primarily activate lateral parietal areas, with the areas of (co)activation spreading into other parts of the parietal cortex and thus extending into the anterior precuneus. This functional specialization within BA 7m could also reflect underlying cytoarchitectonical differences. Based on gradual rostrocaudal architectonic changes within area 7, Brodmann [72] described two main subdivisions, which he named 7a and 7b. A few years later Von Economo and Koskinas [73] described a virtually identical location for their area PE, which was subdivided into the anterior area PEm, with a more pronounced magnocellular appearance, and the relatively smaller-celled posterior area PEp. Topographical comparisons have suggested that PEm and PEp are probably equivalent to Brodmann's subdivisions 7a and 7b [1].

Both the anterior portion of the precuneus (BA 31) and the posterior cingulate cortex (BA 23) are characterized by a rostro-caudal gradient in functional connections. The dorsal portion seems to be functionally associated with the TPN, whereas the ventral portion, along with the retrosplenial cortex (BA 29 and BA 30), is selectively interlinked with the TNN. As reported by Fox at al. [5], [74], the TPN includes pre-supplementary motor area, IPS, FEF, right insular cortex and DLPFC and activates during performance of externally directed cognitively demanding tasks. On the other hand, the TNN includes medial prefrontal cortex, posterior cingulate/precuneus, and angular gyrus, and activates during self-reflective tasks.

A review of the literature in terms of PCC duality suggests that the dorsal anterior portion plays a role within the TPN in orienting the body in space via the cingulate motor areas, whereas the ventral portion interacts with subgenual cortex and other components of the TNN to process self-relevant emotional and non-emotional information and objects and self-reflection [75]. Moreover, the TNN shows considerable overlap with the DMN, thus supporting the hypothesis that the ventral anterior PMC might contribute to resting-state functions such as episodic memory retrieval and reflective awareness. Our results confirm previous findings from both functional connectivity studies in humans [2] and tracing experiments in primates [19] showing selective interconnectivity between the PCC and the parahippocampal formation in subserving episodic memory retrieval. On the other hand, the functional significance of the specific connectivity pattern between the PMC and the claustrum, a neuronal structure which is interconnected with almost all cortical regions, is still unclear [66], [76], [77].

In our study, the retrosplenial cortex (BA 29 and BA 30) was characterized by selective functional correlations with the medial aspect of the temporal lobe and a number of subcortical structures, including the amygdala, the left nucleus accumbens, the left claustrum, and the caudate (left: positive correlation; right: negative correlation), and the dorsomedial thalamus (both positive and negative correlations). Again, these results replicate with fair accuracy the connectivity patterns observed in the primate brain [19]. This functional pattern is also consistent with the known cytoarchitectonical similarities between the retrosplenial allocortex and limbic structures [75]. In addition to its putative contribution to emotional processing networks [78], the retrosplenial cortex is the main source of visuo-spatial information to the mTL memory system. The strong connections with the parahippocampal cortex are of particular importance because they are thought to be related with co-activation of the two regions during spatial navigation tasks. Recent findings indicate that the parahippocampal cortex and retrosplenial cortex have distinct and complementary roles in spatial cognition, with the parahippocampal cortex more concerned with representation of the local visual scene and RSC more concerned with situating the scene within the broader spatial environment [79]. Moreover, it has recently been proposed that the retrosplenial cortex works together with the parieto-occipital sulcus allowing the integration between allocentric environmental representations provided by the hippocampus and mTL and parietal egocentric representations [80].

The emerging picture suggests that the ventral anterior portion of the PMC plays a pivotal role within the DMN in self-referential processing (including self-awareness and autobiographical/episodic memory retrieval) along with the TNN areas which are active during the conscious resting state. The retrosplenial cortex could contribute to this “default” processing by contributing to emotional salience attribution in conjunction with its limbic connections. It might also take part in visuo-spatial attention shifting, along the anterior portion of the PMC, which is an integral part of the TPN.

### Comparison with previous studies

A recent study by Margulies et al. [20] investigating the functional connectivity of the posteromedial cortex in humans and macaque monkeys provided very similar results to our study but also some relevant differences. Interestingly, the subdivision of the PMC in four areas is a common result of both studies. However, the portion of the posteromedial parietal cortex that is connected to the task-positive/cognitive network is located in the most anterior section of the PCC (BA 23) according to the present study, whilst the findings from the study by Margulies et al. locate a similar network in a more posterior section of the precuneus (BA 7) (see Supporting file S2). This discrepancy of ROIwise parcellation may in our opinion be due to a number of methodological factors. First, the studies of Margulies et al. used a larger number of ROIs and a slightly different ROI positioning. For example, ROIs 14 and 15 correspond to ROI 7 in the present study. Second, the concept of “task positive network” is slightly different from the “cognitive network” discussed by Margulies et al. Third, the TPN connectivity pattern shows two separate activations in the PMC (see Supporting file S2), one more anterior (in the BA 23) and one more posterior (in the BA 31). Both our parcellation systems (ROIwise and voxelwise) clustered the anterior activation as TPN proper and the posterior activation as TNN. Different clustering techniques and different datasets/preprocessing strategies may explain why these two areas, which are characterized by a mixed pattern of connectivity, may be clustered in two different ways.

A similar explanation can account for another major discrepancy between the two studies: in our study we found a cluster with connectivity to the visual system that is more posterior/dorsal to the cluster found by Margulies et al. If we compare our results with the results of the 4-clusters ROIwise spectral clustering by Margulies et al. (see Supporting file S2), we see that in this case the differences are minimal. Again, the different clustering strategies might have affected the interpretation of the functional roles of ROIs showing mixed connectivity patterns.

### Conclusions

In summary, our study provides further support for the co-existence of functional unity and diversity within the PMC proposed by Parvizi et al. [19]. First, we showed that there is a considerable degree of functional unity within the PMC, as reflected by strong local interconnections among its components and shared connections with a wide range of other neural structures. However, we also provided evidence for the following distinct functional modules operating within the PMC: (1) the dorsal posterior portion of the precuneus is functionally interconnected with the lateral parietal cortex, motor and premotor cortex; (2) activity in the anterior portion of the precuneus (BA 31) and the posterior cingulate cortex (BA 23) selectively correlates with activity in the TPN (dorsal anterior PMC) and the TNN (ventral anterior PMC); (3) activity in the retrosplenial cortex (BA 29 and BA 30) selectively correlates with activity in posterior mesolimbic structures, including the amygdala and parahippocampal cortex; (4) activity in the ventral part of BA7 selectively correlates with activity in occipital and posterior temporal cortices. This shows that, in addition to shared networks, each area within the PMC has idiosyncratic connectivity patterns with both cortical and subcortical structures involved in different higher association processes. The significant overlap between connectivity patterns and functional correlations suggests that our map of PMC networks could serve as a useful reference tool for future studies aimed at systematically exploring the behavioral correlates of the PMC, and further delineating the impact of task performance activity patterns observed at rest.

## Supporting Information

File S1 Supporting Methods
(0.44 MB PDF)

File S2 Supporting results and discussion
(15.13 MB PDF)
