# Supplementary material for: Functional Connectivity of the Posteromedial Cortex
Source: PLoS One. 2010 Sep 30;5(9):e13107. doi: 10.1371/journal.pone.0013107 (PMC2948030; doi:10.1371/journal.pone.0013107)
Supplement: File S1 — Supporting Methods (0.44 MB PDF) [file pone.0013107.s001.pdf]

Supplementary methods

Preprocessing of the Anatomical Data

Cortex segmentation

In order to perform a cortex-based data analysis, the gray/white matter boundary was segmented using largely automatic segmentation routines [1]. Following the correction of inhomogeneities of signal intensity across space, the white/gray matter border was segmented with a region-growing method using an analysis of intensity histograms. Morphological operations were used to smooth the borders of the segmented data and to separate the left from the right hemisphere. Each segmented hemisphere was finally submitted to a “bridge removal” algorithm, which ensures the creation of topologically correct mesh representations [1]. The borders of the two resulting segmented subvolumes were tessellated to produce a surface reconstruction of the left and right hemisphere. With a fast, fully automatic 3D morphing algorithm [2], the resulting meshes were transformed into inflated and flattened cortex representations. The original folded cortex meshes were used as the reference meshes for projecting connecticity and clustering maps on inflated representations.

High-resolution intersubject cortex alignment

To avoid errors in the intersubject registration a high-resolution, multiscale cortical alignment [3] approach has been used. Since the curvature of the cortex reflects the gyral/sulcal folding pattern of the brain, this brain matching approach essentially aligns corresponding gyri and sulci across subject’s brains. The implemented high-resolution, multiscale cortex alignment procedure has been proven to substantially increase the statistical power and spatial specificity of group analyses [4]. Cortex-based alignment operates in several steps. The folded, topologically correct, cortex representation of each hemisphere constitute the input of the alignment procedure. In the first step, each folded cortex representation is morphed into a spherical representation , which provides a parameterizable surface well suited for across-subject nonrigid alignment. Each vertex on the sphere (spherical coordinate system) corresponds to a vertex of the folded cortex (Cartesian coordinate system) and vice versa. The curvature information computed in the folded representation

is preserved as a curvature map on the spherical representation. The curvature information (folding pattern) is smoothed along the surface to provide spatially extended gradient information driving intercortex alignment minimizing the mean squared differences between the curvature of a source and a target sphere. The essential step of the alignment is an iterative procedure following a coarse-to-fine matching strategy. Alignment starts with highly smoothed curvature maps and progresses to only slightly smoothed curvature representations. Starting with a coarse alignment as provided by AC-PC or Talairach space, this method ensures that the smoothed curvature of the two cortices possess enough overlap for a locally operating gradient-descent procedure to converge without user intervention [3]. Visual inspection and a measure of the averaged mean squared curvature difference reveal that the alignment of major gyri and sulci can be achieved reliably by this method. To define a target brain for alignment. we employed the "moving target" approach: a "moving target" computed repeatedly during the alignment process as the average curvature across all hemispheres at a given alignment stage is then created. The procedure starts with the coarsest curvature maps. Then the next finer curvature maps are used and averaged with the obtained alignment result of the previous level. The established correspondence mapping between vertices of the cortices is used to align the subjects' gray matter maps, connectivity and clustering maps.

Correction for multiple comparisons

In this paper we used a recently implemented approach based on a 3D extension of the randomization procedure described in Forman et al. [5] for multiple comparison correction as suggested by Goebel et al. in [6]. First, a voxel-level threshold was set at t correspondent to a $P < 0.01$, uncorrected. Thresholded maps were then submitted to a whole-brain correction criterion based on the estimate of the map's spatial smoothness and on an iterative procedure (Monte Carlo simulation) for estimating cluster-level false-positive rates. After 2000 iterations, the minimum cluster size threshold that yielded a cluster-level false-positive rate (alpha) of 5% was applied to the statistical maps. The implemented method corrects for multiple cluster tests across space. For each simulated image, all "active" clusters in the imaged volume are considered and used to update a table reporting the counts of all the clusters above this threshold for each specific size. After a suitable number of iterations (e.g., 2000), an alpha value is assigned to each cluster size based on its observed relative frequency. From this information the minimum cluster size threshold was specified in order to yield a cluster-level false-positive rate of 5%.

Clustering

Voxelwise clustering: Fuzzy clustering

Fuzzy clustering attempts to partition a subset of N voxels in C 'clusters' of activation [7]. This is achieved by comparing the voxel's time courses $\mathbf{x}_n$ (n=1…N) with each other and assigning them to representative time courses, called cluster centroids $\mathbf{v}_c$ (c=1…C), derived during this process. Fuzziness relates to the fact that a voxel is generally not uniquely assigned to one cluster only (hard clustering), but instead, the similarity of the voxel time course to each cluster centroid is determined. This is expressed by the 'membership' u_{cn} of voxel n to cluster c. For each voxel, we have:

$$\sum_{c=1}^{C} u_{cn} = 1 \quad (1)$$

Both centroids $\mathbf{v}_c$ and memberships u_{cn} are updated in an iterative procedure, elaborated by Bezdek et al. [8] and expressed by:

$$\mathbf{v}_c = \frac{\sum_{n=1}^{N} u_{cn}{}^{m}\mathbf{x}_n}{\sum_{n=1}^{N} u_{cn}{}^{m}} \qquad u_{cn} = \frac{1}{\sum_{k=1}^{C}\left(\frac{d(\mathbf{x}_n,\mathbf{v}_c)}{d(\mathbf{x}_n,\mathbf{v}_k)}\right)^{\frac{2}{m-2}}} \quad (2)$$

where d is a distance measure, determining the similarity between the time course of a voxel and a cluster center, and m is the fuzziness coefficient, determining the fuzziness of the procedure and used to 'tune out' the noise in the data. Theoretically, m lies between 1 (smallest fuzziness) and infinity. Its ideal value, however, is problem dependent. Several distance measures d can be defined: the Euclidean distance d_{E} and the Mahalanobis distance d_{M} [9] are mostly used and are defined as:

$$d_{\mathrm{E}}(\mathbf{x}_n,\mathbf{v}_c)=\|\mathbf{x}_n-\mathbf{v}_c\|^2 \quad (3)$$

$$d_{\mathrm{M}}(\mathbf{x}_n,\mathbf{v}_c) = (\mathbf{x}_n - \mathbf{v}_c)^{\mathsf{T}}\mathrm{R}_c^{-1}(\mathbf{x}_n - \mathbf{v}_c) \quad (4)$$

where Σ_c represents the covariance matrix of cluster c. The Mahalanobis distance takes into account the actual (elliptical) shape of the cluster, i.e., instead of treating all voxels $\mathbf{x}_n$equally when calculating the distance d to the cluster centre $\mathbf{v}_c$, it weights the differences by the range of variability, described by Σ_c, in the direction of the voxel. The Euclidean distance does not take into account the shape of the cluster, i.e., it assumes a spherical shape, corresponding to a covariance matrix Σ_c with 1s on the main diagonal and 0s elsewhere.

The algorithm starts from an initial set of membership values for the data set, expressed in matrix form as:

$$U^{(0)} = \left(1 - \frac{\sqrt{2}}{2}\right)U + \frac{\sqrt{2}}{2}V \quad (5)$$

with U=1/C and V a matrix of randomly chosen cluster centres. Next, the new cluster centres and memberships are computed using Eq. (2). The procedure terminates when successive iterations do not further change significantly memberships and cluster centres, as calculated by Eq. (2). This procedure corresponds to the minimization of the following objective function:

$$\sigma_{\mathrm{w}}^2 = \frac{1}{N}\sum_{n=1}^{N}\sum_{c=1}^{C} u_{cn}^m d(\mathbf{x}_n, \mathbf{v}_c) \quad (6)$$

which computes the within-class variance over all clusters σ_{w}^2. In practice, a user-defined threshold for change in σ_{w}^2 determines when convergence is reached. Preprocessing includes the transformation of each time series into its z-score so as to avoid the clustering algorithm to classify the voxels based on signal amplitude, instead of signal shape. Finally, PCA is performed to reduce data dimensionality.

Voxelwise clustering: optimal number of clusters

The a priori determination of the fuzziness coefficient and the number of clusters are research topics often encountered in the literature [10]. Critically, the “true” number of clusters (i.e. optimal number of classes) is usually unknown in Fuzzy clustering. In this perspective, several cluster-validity indices have previously been proposed in the literature to appreciate, in an unsupervised manner, the optimal number of clusters (for a review see [11]). These indices combined different measures of compactness and separation of the clustering in order to ensure that identified clusters are compact and well-separated. In our paper, we used two different methods: (i) a cross-validation method: the group was split in half, and Jaccard's J (which measures *dis*similarity between sample sets is obtained by dividing the difference of the sizes of the union and the intersection of two sets by the size of the union:

$$J_\delta(A,B) = 1 - J(A,B) = \frac{|A \cup B| - |A \cap B|}{|A \cup B|}. \tag{7}$$

was used to compare clustering solutions across the groups. The consistency check was performed for between 2 and 10 clusters, the minimum number of clusters that minimize the Jaccard dissimilarity index J was choosen. It yielded local maxima of 4-cluster solution. (ii) Using the similarity index generated by the SogIca method (see [12]) we choose the minimum number of clusters that maximized the combined similarity index for each clustered group. The similarity index here employed is the absolute value of the mutual correlation coefficients, in space for the spatial sources of estimates or in time for the associated basis time-courses; this measure give a combined value of similarity based on spatial and temporal correlation (see [12]).

Group components clusterization

To obtain a unsupervised group components clusterization of all the single subject clusters generated by the Fuzzy clustering technique we employed the Self organizing group ICA. This method [12], originally developed for single subject ICA results can be successfully employed also for our data, indeed with this method the clusters of single-subject decompositions are grouped according to the combined spatio-temporal information using a self organizing grouping procedure that is based on hierarchical cluster analysis [13].

Voxel distance calculations

For each ROI we calculated the Euclidean distance between the center of the ROI and every other voxel that reached significance in the thresholded Z-score map of positive FC (cluster significance: $p < 0.05$). The Euclidian distance between two voxels $P = (p_x, p_y, p_z)$ and $Q = (q_x, q_y, q_z)$ was computed with the formula $\sqrt{}\ (p_x - q_x)^2 + (p_y - q_y)^2 + (p_z - q_z)^2$. Then, for each ROI, we computed the number of significant voxels at specific distances (from 0 to 140 mm in 4-mm bins) from the center of the ROI for each individual. Such ranges thus comprise short- (< 40 mm), medium- (40-80 mm) and long- (> 80 mm) distance connections.

Matlab Scripting

We created a Maltab® script (version 7.0) for voxel of interest (VOI) analysis. Using the AFNI data collection (http://afni.nimh.nih.gov/afni/doc/misc/afni_ttatlas), we saved two atlases, each consisting of arrays of 140x172x120 voxels with a 1x1x1 mm^3 resolution, in .mat format. The first was created for Gyrus classification of the normalized brain in the Talairach space (Fig A1) and the second for Brodmann Areas (Fig A2).

Fig A1. Gyrus AFNI Template

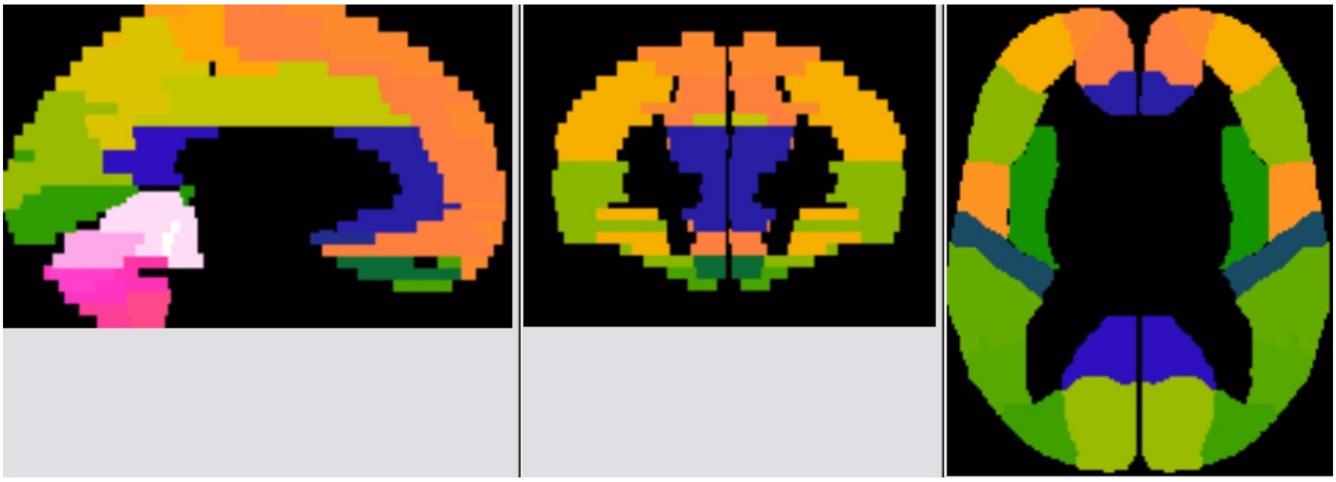

Three orthogonal slices of the template showing different Gyri in different colors

We then created other atlases forcing the classification of null voxels on the basis of a simple algorithm: if the absolute majority (>0.5) of nearest voxels (see Fig A3 bottom left) belong to a category we assigned that voxel to it. Using the algorithm iteratively we saved eight more atlases (called R1, R2, R3, R4) with an increasing number of classified voxels, but also with some image degradation (see Fig A3).

Fig A2. Brodmann Areas AFNI Template

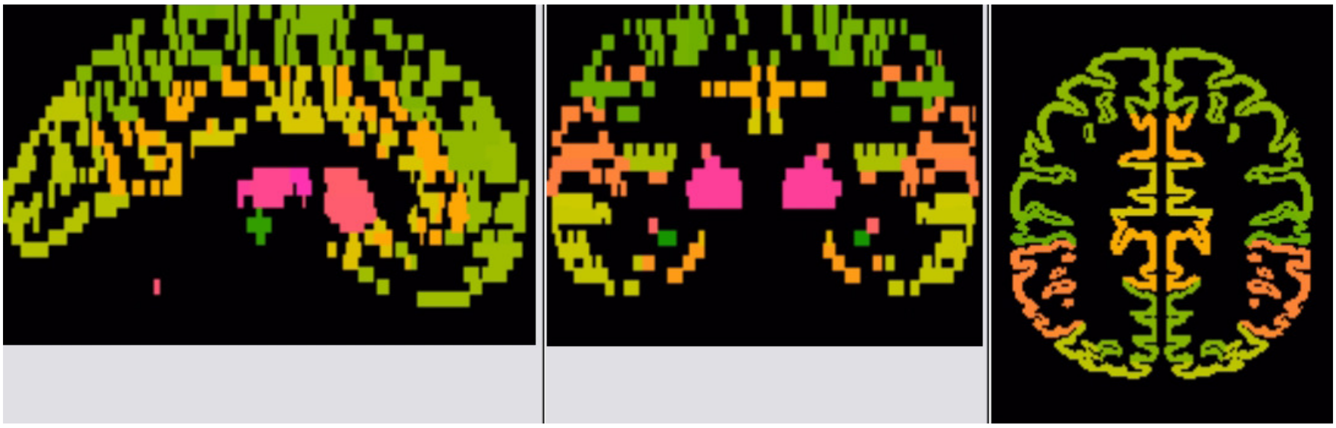

Three orthogonal slices of the template showing different BAs in different colors

The inputs of the script were .voi files saved from BrainVoyager QX volume maps. These files contained samples of statistically significant voxels, divided into clusters. The script can compare these with the selected atlases to produce masked subsamples of the voxels with respect to a prespecified VOI.

Fig A3. Brodmann Area AFNI R1 Template

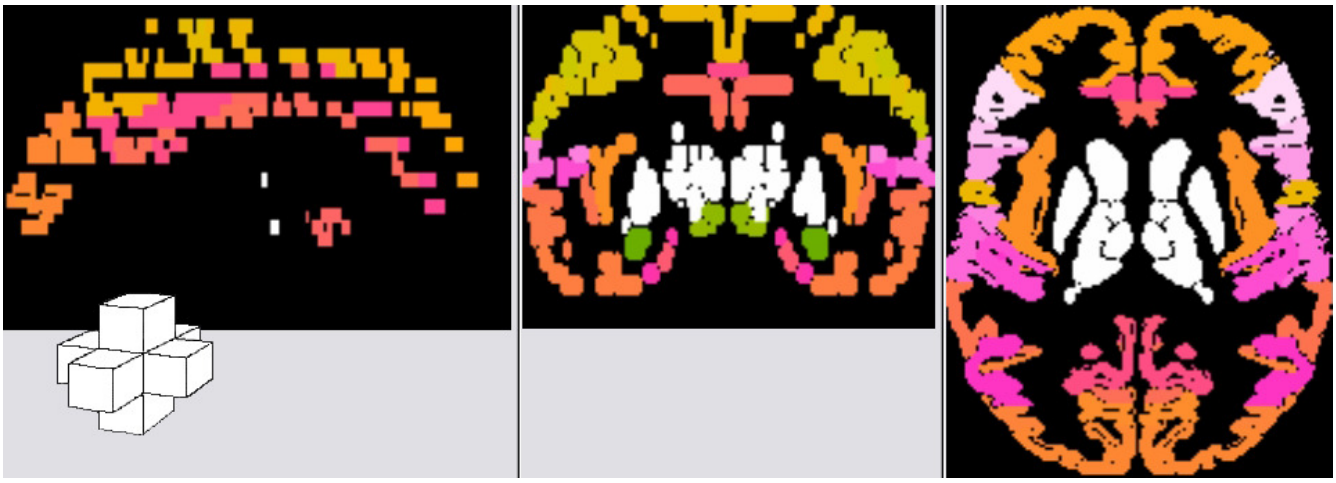

Three orthogonal slices of the template showing different BAs in different colors after near voxel forced classification

The Gyrus/BA output of the script produces three graphs using the selected AFNI Gyrus or BA atlas:

The percentage of active voxels divided by the cerebral gyri (see Fig A4 on the left) or BA, the procedure stops at a fixed threshold of the total number of voxels (p.e. with a 5% threshold the

script graphs the gyri that contain a number of voxel greater than 5% of total voxel, see Fig A4)
The lateralization percent of the gyri or BA that overcome the threshold (see Fig A4 on the right).
The number of active voxels of the supra-threshold areas as a function of the gyrus or BA and of the Talairach coordinate X (left to right, see Fig A5).

Fig A4. Gyrus Output 1 & 2

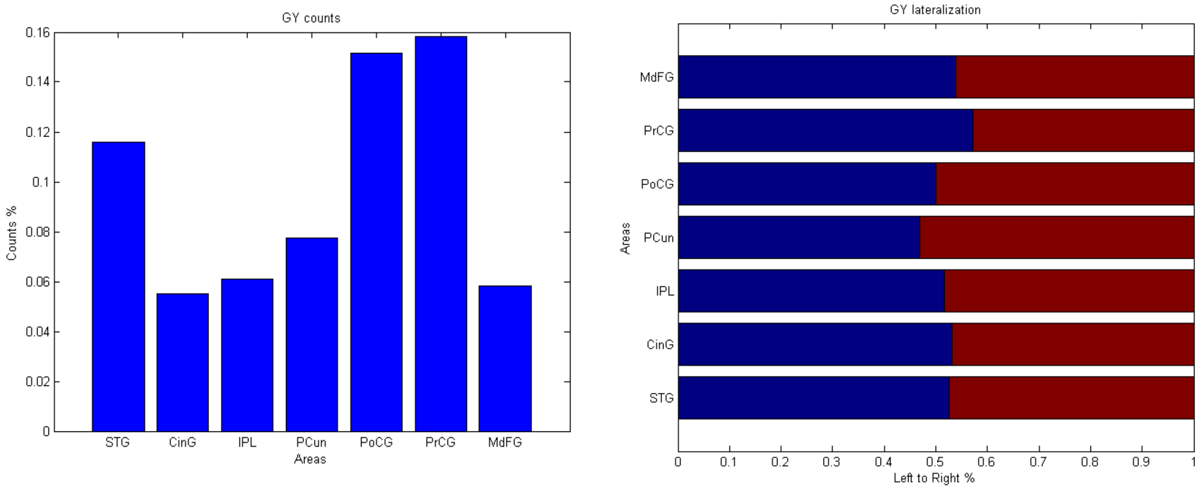

Gyrus counts percent (left) and gyrus lateralization (right)

Fig A5. Gyrus Output 3

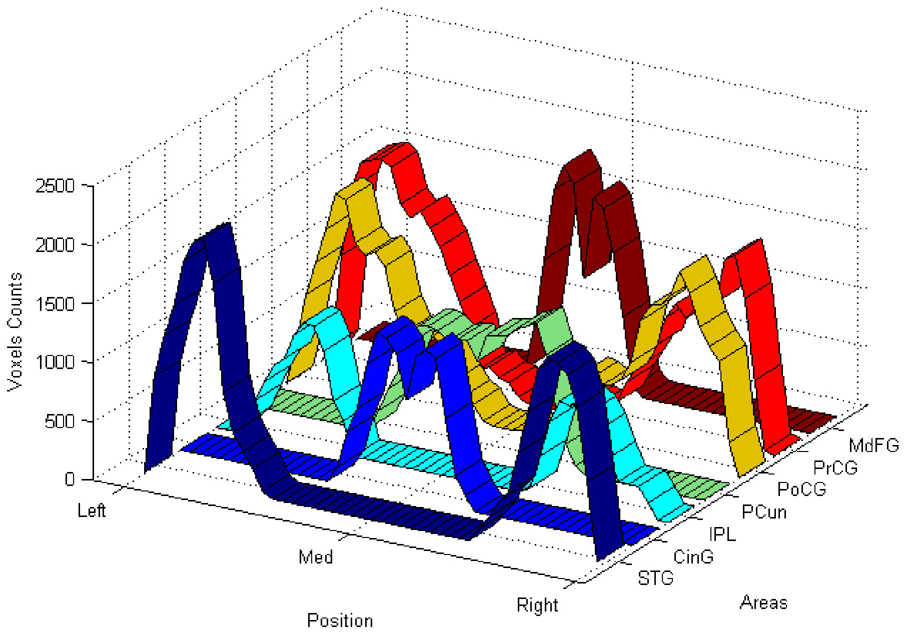

Gyrus counts in function of areas and lateralization

The possible abbreviations of the gyrus output are: PCC = Posterior Cingulate; ACC = Anterior Cingulate; SbCG = Subcallosal Gyrus; TTG = Transverse Temporal Gyrus; Unc = Uncus; RG = Rectal Gyrus; FG = Fusiform Gyrus; IOG = Inferior Occipital Gyrus; ITG = Inferior Temporal Gyrus; Ins = Insula; PaHG = Parahippocampal Gyrus; LG = Lingual Gyrus; MOG = Middle Occipital Gyrus; OrG = Orbital Gyrus; MTG = Middle Temporal Gyrus; STG = Superior Temporal Gyrus; SOG = Superior Occipital Gyrus; IFG = Inferior Frontal Gyrus; Cun = Cuneus; Ang = Angular Gyrus; SMG = Supramarginal Gyrus; CinG = Cingulate Gyrus; IPL = Inferior Parietal Lobule; Pcun = Precuneus; SPL = Superior Parietal Lobule; MFG = Middle Frontal Gyrus; PaCL = Paracentral Lobule; PoCG = Postcentral Gyrus; PrCG = Precentral Gyrus; SFG = Superior Frontal Gyrus; MdFG = Medial Frontal Gyrus; vUv = Uvula of Vermis; vPyr = Pyramis of Vermis; vTub = Tuber of Vermis; vDec = Declive of Vermis; vCul = Culmen of Vermis; Cton = Cerebellar Tonsil; SLun = Inferior Semi-Lunar Lobule; Fast = Fastigium; Dent = Dentate; Nod = Nodule; Uvu = Uvula; Pyr = Pyramis; Tub = Tuber; Dec = Declive; Cul = Culmen; Clin = Cerebellar Lingual.

The possible abbreviations of the BA are: Hippo = Hippocampus; Amg = Amygdala; HyTH = Hypothalamus; SN = Substantia Nigra; CauTa = Caudate Tail; CauBo = Caudate Body; CauHd = Caudate Head; VAN = Ventral Anterior Nucleus; VPMN = Ventral Posterior Medial Nucleus; VPLN = Ventral Posterior Lateral Nucleus; MDN = Medial Dorsal Nucleus; LDN = Lateral Dorsal Nucleus; Pulv = Pulvinar; LPN = Lateral Posterior Nucleus; VLN = Ventral Lateral Nucleus; MN = Midline Nucleus; AN = Anterior Nucleus; MaBo = Mammillary Body; Md GP = Medial Globus Pallidus; Lt GP = Lateral Globus Pallidus; Put = Putamen; NAcc =Nucleus Accumbens; MGB =

Medial Geniculum Body; LGB = Lateral Geniculum Body; SuTH = Subthalamic Nucleus; BA 1-47 = Brodmann Area 1-47.

The Table output of the script is a .txt file with many rows. Every row represents an area that meet some criteria: the number of active voxels included in the area surpasses a fixed fraction of total activation voxels (default = 5%) and/or the number of active voxels surpasses a fixed fraction of the total voxels of that area (default = 25%) and/or the number of active voxels surpasses a fixed absolute number of voxels in subcortical areas (default = 125) and the number of active voxels surpasses a minimum fixed absolute number of voxels (default = 100).

Every row contains the following data: name of the gyrus preceded by the left, right or bilateral attribute, number of active voxels, lateralization, Brodmann areas of the voxels group in decrescent order. The maximum number of Brodmann Areas written in the table is fixed (default = 4) not to obscure the output readability.

The script and the atlases (both in .mat or .img format) are available to anyone for examination or use, if interested, please send an email at federico.dagata@unito.it

REFERENCES

1. Kriegeskorte N, Goebel R (2001) An efficient algorithm for topologically correct segmentation of the cortical sheet in anatomical mr volumes. Neuroimage 14: 329-346.
2. Goebel R (2000) A fast automated method for flattening cortical surfaces. Neuroimage 11: S680.
3. Goebel R, Hasson U, Lefi I, Malach R (2004) Statistical analyses across aligned cortical hemispheres reveal high-resolution population maps of human visual cortex. Neuroimage 22.
4. van Atteveldt N, Formisano E, Goebel R, Blomert L (2004) Integration of letters and speech sounds in the human brain. Neuron 43: 271-282.
5. Forman SD, Cohen JD, Fitzgerald M, Eddy WF, Mintun MA, et al. (1995) Improved assessment of significant activation in functional magnetic resonance imaging (fMRI): use of a cluster-size threshold. Magn Reson Med 33: 636-647.
6. Goebel R, Esposito F, Formisano E (2006) Analysis of functional image analysis contest (FIAC) data with brainvoyager QX: From single-subject to cortically aligned group general linear model analysis and self-organizing group independent component analysis. Hum Brain Mapp 27: 392-401.
7. Zadeh LA (1977) Fuzzy Set and Their Application to Pattern Recognition and Clustering Analysis. In: Van Ryzin J, editor. Classification and clustering : proceedings of an Advanced Seminar conducted by the Mathematics Research Center, the University of Wisconsin at Madison, May 3-5, 1976. New York ; London: Academic Press. pp. 355-393.
8. Bezdek JC, Ehrlich R, Full W (1984) FCM: The fuzzy c-means clustering algorithm. Computers & Geosciences 10: 191-203.
9. Mahalanobis PC (1936) On the generalised distance in statistics. Proc Natl Acad Sci U S A 2.
10. Fadili MJ, Ruan S, Bloyet D, Mazoyer B (2001) On the number of clusters and the fuzziness index for unsupervised FCA application to BOLD fMRI time series. Med Image Anal 5: 55-67.
11. Wang W, Zhang Y (2007) On fuzzy cluster validity indices. Fuzzy Sets Sys 158: 2095-2117.
12. Esposito F, Scarabino T, Hyvarinen A, Himberg J, Formisano E, et al. (2005) Independent component analysis of fMRI group studies by self-organizing clustering. Neuroimage 25: 193-205.

13. Himberg J, Hyvarinen A, Esposito F (2004) Validating the independent components of neuroimaging time series via clustering and visualization. Neuroimage 22: 1214-1222.
14. Fair DA, Cohen AL, Dosenbach NU, Church JA, Miezin FM, et al. (2008) The maturing architecture of the brain's default network. Proc Natl Acad Sci U S A 105: 4028-4032.
15. Stevens MC, Pearlson GD, Calhoun VD (2009) Changes in the interaction of resting-state neural networks from adolescence to adulthood. Hum Brain Mapp.
16. Greicius MD, Krasnow B, Reiss AL, Menon V (2003) Functional connectivity in the resting brain: a network analysis of the default mode hypothesis. Proc Natl Acad Sci U S A 100: 253-258.
17. Raichle ME, MacLeod AM, Snyder AZ, Powers WJ, Gusnard DA, et al. (2001) A default mode of brain function. Proc Natl Acad Sci U S A 98: 676-682.
18. Raichle ME, Snyder AZ (2007) A default mode of brain function: a brief history of an evolving idea. Neuroimage 37: 1083-1090; discussion 1097-1089.
19. Fox MD, Snyder AZ, Vincent JL, Corbetta M, Van Essen DC, et al. (2005) The human brain is intrinsically organized into dynamic, anticorrelated functional networks. Proc Natl Acad Sci U S A 102: 9673-9678.
20. De Luca M, Beckmann CF, De Stefano N, Matthews PM, Smith SM (2006) fMRI resting state networks define distinct modes of long-distance interactions in the human brain. Neuroimage 29: 1359-1367.
21. Fransson P (2006) How default is the default mode of brain function? Further evidence from intrinsic BOLD signal fluctuations. Neuropsychologia 44: 2836-2845.
22. Shehzad Z, Kelly AM, Reiss PT, Gee DG, Gotimer K, et al. (2009) The Resting Brain: Unconstrained yet Reliable. Cereb Cortex.
23. Wu CW, Gu H, Lu H, Stein EA, Chen JH, et al. (2009) Mapping functional connectivity based on synchronized CMRO2 fluctuations during the resting state. Neuroimage 45: 694-701.
24. Damoiseaux JS, Rombouts SA, Barkhof F, Scheltens P, Stam CJ, et al. (2006) Consistent resting-state networks across healthy subjects. Proc Natl Acad Sci U S A 103: 13848-13853.
25. Luo L, O'Leary DD (2005) Axon retraction and degeneration in development and disease. Annu Rev Neurosci 28: 127-156.
26. Fox MD, Raichle ME (2007) Spontaneous fluctuations in brain activity observed with functional magnetic resonance imaging. Nat Rev Neurosci 8: 700-711.
27. Margulies DS, Vincent JL, Kelly C, Lohmann G, Uddin LQ, et al. (2009) Precuneus shares intrinsic functional architecture in humans and monkeys. Proc Natl Acad Sci U S A 106: 20069-20074.

28. Goebel R, Roebroeck A, Kim DS, Formisano E (2003) Investigating directed cortical interactions in time-resolved fMRI data using vector autoregressive modeling and Granger causality mapping. Magn Reson Imaging 21: 1251-1261.
29. Roebroeck A, Formisano E, Goebel R (2005) Mapping directed influence over the brain using Granger causality and fMRI. Neuroimage 25: 230-242.
