# Supplementary material for: Functional Connectivity of the Posteromedial Cortex
Source: PLoS One. 2010 Sep 30;5(9):e13107. doi: 10.1371/journal.pone.0013107 (PMC2948030; doi:10.1371/journal.pone.0013107)
Supplement: File S2 — Supporting results and discussion (15.13 MB PDF) [file pone.0013107.s002.pdf]

Supplementary Results

Although we used age and gender as covariate in the second level statistics, we further inspected possible age or gender effects on the rsFC maps performing a correlational analysis between each subject-specific ROI-generated map, age and gender using the ANCOVA analysis tool implemented in BrainVoyager QX. We found no significant correlation between them ($q<0.05$ FDR corrected, cluster threshold $K>5$ voxels in the native resolution). This finding can be viewed as in opposition with previous works [14,15] in which an age–related decrease in the DMN connectivity is found. A possible explanation is that age-related effects using correlational methods are better visualized with bigger groups; moreover with a less stringent threshold ($P<0.05$ uncorrected) some age and sex-related connectivity patterns emerged also in our data (data not shown).

Movement was assessed by summing the deviations (3 translations plus 3 rotations at a radius of 50 mm) used to compensate for head motion within fMRI. The presently reported quantity is head movement rms mm averaged over subjects. This quantity was 0.29 ± 0.09 mm (mean ± standard deviation) for the 16 subjects. Pearson bivariate coefficients were calculated with SPSS 13.0 for movement and age. The result was 0.09 ($P = 0.75$), thus suggesting that it is unlikely that subjects' age correlated with head movements in the MR scanner.
Inspecting the probabilistic map showing the conjunction of all the ROI-related connectivity pattern together, we observed that the positively correlated areas showed a higher spatial overlapping than the negatively correlated areas. Areas found to be more frequently positively correlated are the bilateral posterior cingulate cortex/precuneus (60% of the ROI connected), cuneus (50% of the ROI), lingual gyrus (30% of the ROI), angular gyrus (50% of the ROI), postcentral gyri (BA 43) (30% of the ROI), middle frontal gyrus (BA 6) (30% of the ROI), postcentral gyrus (BA 2) (30% of the ROI), motor-supplementary motor cortex (40% of the ROI), amygdala (20% of the ROI), inferior parietal lobule (40% of the ROI), dorsolateral prefrontal cortex (DLPFC) (40% of the ROI), right anterior insula (26% of the ROI), cerebellar hemispheres (40% of the ROI) and ventromedial prefrontal cortex (VMPFC) (40% of the ROI). (See Fig 4, left and middle panels, and Fig S1) Areas found to be more often anticorrelated are the bilateral posterior cingulated cortex/precunei (29% of the ROI), right dorsal precuneus (26% of the ROI), middle frontal gyrus (BA 10) (19% of the ROI) and right supramarginal gyrus (26% of the ROI).

In Tab S3 for each ROI positive and negative correlations with the main cortical regions are summarized. As expected, different ROIs have a similar pattern of correlations and most of the cortical regions involved are part of the Default Mode Network (DMN) [16,17,18].

Recently, Fox et al. [19] proposed the analysis of connectivity in the resting human brain in term of two diametrically opposed brain networks, identified on the basis of both spontaneous correlations within each network and anticorrelations between networks; the authors have identified cortical foci for intrinsically defined anticorrelated networks, the task-positive network (TPN) and the task-negative network (TNN). Although some areas have a dominant connectivity to other networks (eg Visual or Motor) almost all different sub-regions of PMC are involved in one of those two networks. Therefore we have also analyzed the correlation of ROIs of PMC with respect to the two networks. In Table S4 we have plotted the correlations of each ROI with the peak foci identified by Fox et al. [19]. Within this conceptual framework, we found that ROI 1, 5, 6, 8 and 10 are related to TPN; on the contrary, ROI 2, 3, 4, 7, 9 are related to TNN. More in detail, both ROI 8 and 10 are anticorrelated (negative correlations) with TNN; on the contrary, both ROI 3 and 9 are anticorrelated with TPN (See Fig 2b). Moreover, ROI 3 and ROI 9 have positive correlations with prefrontal cortex (BA 10) and have a pivotal role in integration of fronto-parietal network of the DMN.

ROI 3 and ROI 4 have a complex pattern of correlation; in fact, they have relevant anticorrelations with both TPN and TNN, but they are related to TNN: ROI 4 has positive correlations with temporomesial network (parahippocampal cortex and amygdalae) and, therefore, this ROI is related to TNN; ROI 3 has positive correlations with PCC/retrosplenial cortex, MPF and inferior temporal cortex (see Fig 2b and table S3). Therefore, BA 29/30 (ROI 3 and ROI 4) is related to TNN and to the temporal lobe (ROI 4 with limbic mesial temporal cortex, ROI 3 with lateral temporal neocortex).

ROI 5 and ROI 6 have an identical pattern of broad correlations, related to motor and premotor cortex, cerebellum and visual system; therefore we consider them strictly interrelated. Besides, the bilateral SPL/IPL and the FEF, ROI 5 and ROI 1 have a similar pattern of correlation involving motor/premotor cortex and insular cortex (See Tab S4). Our results show interesting differences between these ROIs: ROI 5 is correlated with the sensorimotor system, whereas ROI 1 is anticorrelated with the same network; moreover, ROI 1 has positive correlations with the temporal lobe (temporal network), whereas ROI 5 correlates with frontal cortex (frontal network).

ROI 2 and ROI 7 have similar positive (PCC/IPL and FEF) and negative (MeFC) correlations; these ROIs have a complementary pattern of correlation with ACC (ROI 2 correlates positively, ROI 7 correlates negatively).

Lateralization

Mapping the lateralization on ROI-specific plots, we found that the positively correlated networks are less lateralized than the negatively correlated networks. Indeed, within the positively correlated networks, only ROI 7+ and 8+ have highly lateralized areas (BA 39 in ROI 7+ and BA 7 in ROI 8+), while negatively correlated networks are generally more lateralized (BA 2, 3, 4, 31 are left lateralized in ROI 1; BA 17 and 24 are left lateralized in ROI 2-; BA 39 and 40 are right lateralized in ROI 3; BA 8, 10, 44, 45, 47 and caudate are right lateralized in ROI 4; BA 7, caudate body and pulvinar are right lateralized in ROI 5; ROI 6 BA 6, 9, 39, 40, left lateralized; ROI 7 BA 10, 18 and putamen right lateralized while BA 31 and 7 left lateralized; ROI 8 BA 10 left lateralized; ROI 9 BA 40 left lateralized and finally ROI 10 show BA 6 and 8 left lateralized (See Fig S4-S13 and S15).

Voxel-distance Differences

Fig S4-S13 displays the mean number of significantly positively (yellow bars) and negative (blue bars) correlated voxels at specific distances (from 0 to 140 mm in 4-mm bins) from the center of each ROI (0-40 mm = short distance, 40-96 mm = medium distance, 96-140 mm = long distance).

<u>ROI 1</u>

The greatest number of positively correlated voxels were at medium distance (90415 voxels), followed by short distance (31675 voxels), and long distance (812 voxels).

The greatest number of negatively correlated voxels were at medium distance (14264voxels), followed by short distance (3239 voxels), and long distance (666 voxels).

<u>ROI 2</u>

The greatest number of positively correlated voxels were at short distance (18883voxels), followed by medium distance (6904 voxels), and long distance (0 voxels).

The greatest number of negatively correlated voxels were at short distance (3687 voxels), followed by medium distance (1338 voxels), and long distance (496 voxels).

ROI 3

The greatest number of positively correlated voxels were at long distance (27316 voxels), followed by short distance (25695 voxels), and medium distance (6848 voxels).

The greatest number of negatively correlated voxels were at medium distance (13079 voxels), followed by short distance (3322 voxels), and long distance (1535 voxels).

ROI 4

The greatest number of positively correlated voxels were at short distance (89940 voxels), followed by medium distance (54653 voxels), and short distance (39237 voxels).

The greatest number of negatively correlated voxels were at long distance (8496 voxels), followed by medium distance (5532 voxels), and short distance (1036 voxels).

ROI 5

The greatest number of positively correlated voxels were at medium distance (125789 voxels), followed by short distance (86516 voxels), and long distance (36540 voxels).

The greatest number of negatively correlated voxels were at medium distance (3623 voxels), followed by short distance (1507 voxels), and long distance (548 voxels).

ROI 6

The greatest number of positively correlated voxels were at long distance (56734voxels), followed by short distance (50746 voxels), and medium distance (38794 voxels).

The greatest number of negatively correlated voxels were at short distance (1595 voxels), followed by medium distance (1263 voxels), and long distance (160 voxels).

ROI 7

The greatest number of positively correlated voxels were at short distance (32117 voxels), followed by medium distance (7434 voxels), and long distance (1377 voxels).

The greatest number of negatively correlated voxels were at short distance (3933 voxels), followed by long distance (2869 voxels), and medium distance (2867 voxels).

ROI 8

The greatest number of positively correlated voxels were at short distance (28321 voxels), followed by medium distance (6790 voxels), and long distance (6127 voxels).

The greatest number of negatively correlated voxels were at medium distance (29973 voxels), followed by long distance (25331 voxels), and short distance (2492 voxels).

ROI 9

The greatest number of positively correlated voxels were at medium distance (32326 voxels), followed by short distance (28874 voxels), and long distance (25132 voxels).

The greatest number of negatively correlated voxels were at long distance (19800 voxels), followed by medium distance (11659 voxels), and short distance (4605 voxels).

ROI 10

The greatest number of positively correlated voxels were at long distance (76276 voxels), followed by medium distance (51027 voxels), and long distance (7315 voxels).

The greatest number of negatively correlated voxels were at short distance (5466 voxels), followed by long distance (3142 voxels), and medium distance (481 voxels).

Spatial probabilistic maps

To assess the spatial consistency and reproducibility of seed-generated maps we computed spatial probability maps. We found a high level of spatial overlapping and reliability between each subject's specific ROI-related rsFC map (See Fig S4-S13).). We also computed 2D the spatial probability maps (Fig S16) of the 4 network showed in Fig 4 (TNN, TPN, MOT, VIS) and Fig 6.

Reliability index

The split-half test performed with the Spearman Brown method between each ROI in the two split groups show that our results have a good-to-high reliability (RSB>0.60, mean 0.67) (Tab S5).

Methodological considerations

Spontaneous BOLD coactivations are the fMRI correlates of coherent neuronal oscillations observed in neurophysiological studies. Coherent neuronal oscillations may be either spontaneous or related to specific goals, and synchrony could facilitate the coordination and organization of information processing in the brain across both space and time. On the other hand, anticorrelations might play a fundamental role in differentiating and segregating neuronal networks and processes subserving opposite goals or competing representations (Fox et al., 2009; Weissenbacher et al., 2009; Van Dijk, 2010). The use of a global signal regression as covariate of no interest is still under debate. This procedure is arguably helpful in regressing out physiological noise, however it may generate ambiguous results and has the potential to introduce artificial negative correlations. An additional ROIwise clusterization was performed on anticorrelated networks: it yielded identical results to clusterization performed only on positive or combined positive/negative correlations.

Examination of resting-state data alone has a number of limitations that merit consideration. First, there is no full agreement on how to interpret functional connectivity data, especially because it has been shown that functional connectivity can change during task performance [20,21]. Recent studies have also demonstrated that rsFC patterns do not represent artifacts produced by aliasing of cardiac and respiratory cycles, but are localized in the grey matter and are likely related to ongoing neuronal activity [17,20,22,23]. Moreover [24] these networks are characterized by BOLD signal changes comparable with task-related BOLD changes (up to 3%) which are consistent across individuals and stable across repeated sessions.

Second, resting-state analysis faces the same potential limitations as task-related fMRI studies with regards to inter-individual variability in PMC organization and connectivity. However, the patterns of functional differentiation observed using our seeding approach were reliable and detectable at the individual participant level (See Tab S5 and Fig S2-S11).

Third, our subjects were distributed over a wide age-span: we attempted to take into account the inter-subject variability using a random effect analysis and to reduce the variability induced by age and gender differences by controlling these factors inserting age and sex as covariates in the statistics. We also inspected if subjects' movements were correlated with age and found a small quantity of overall movements and no significant correlation between age and movements, thus suggesting that our results were not significantly influenced by these confounders.

Furthermore, the interpretation of our findings needs caution because the networks described here are detected in the absence of specific functional activity. We are inferring functional roles for the PMC areas based on their belonging to intrinsic connectivity networks, whose functional relevance

is reasonably well established in the literature. Although the exact functional significance of temporal correlations in very low-frequency neural fluctuations remains largely unclear, it has been argued that this basal, task-independent, intrinsic connectivity is important to avoid disuse-related pruning of critical synapses {Luo, 2005 #584} and/or to maintain networks in a primed state, thus improving response efficiency [26].

Finally, although the convergence of our findings with more precise tract-tracing studies in primates and with recent rsFC findings [27] is reassuring, it is worth noting that the BOLD signal used in fMRI is only an indirect measure of neural activity and does not address hierarchy or causality [28,29] within any of the networks identified.

Supplementary Figures

Fig S1. Right and left monolateral ROI comparison

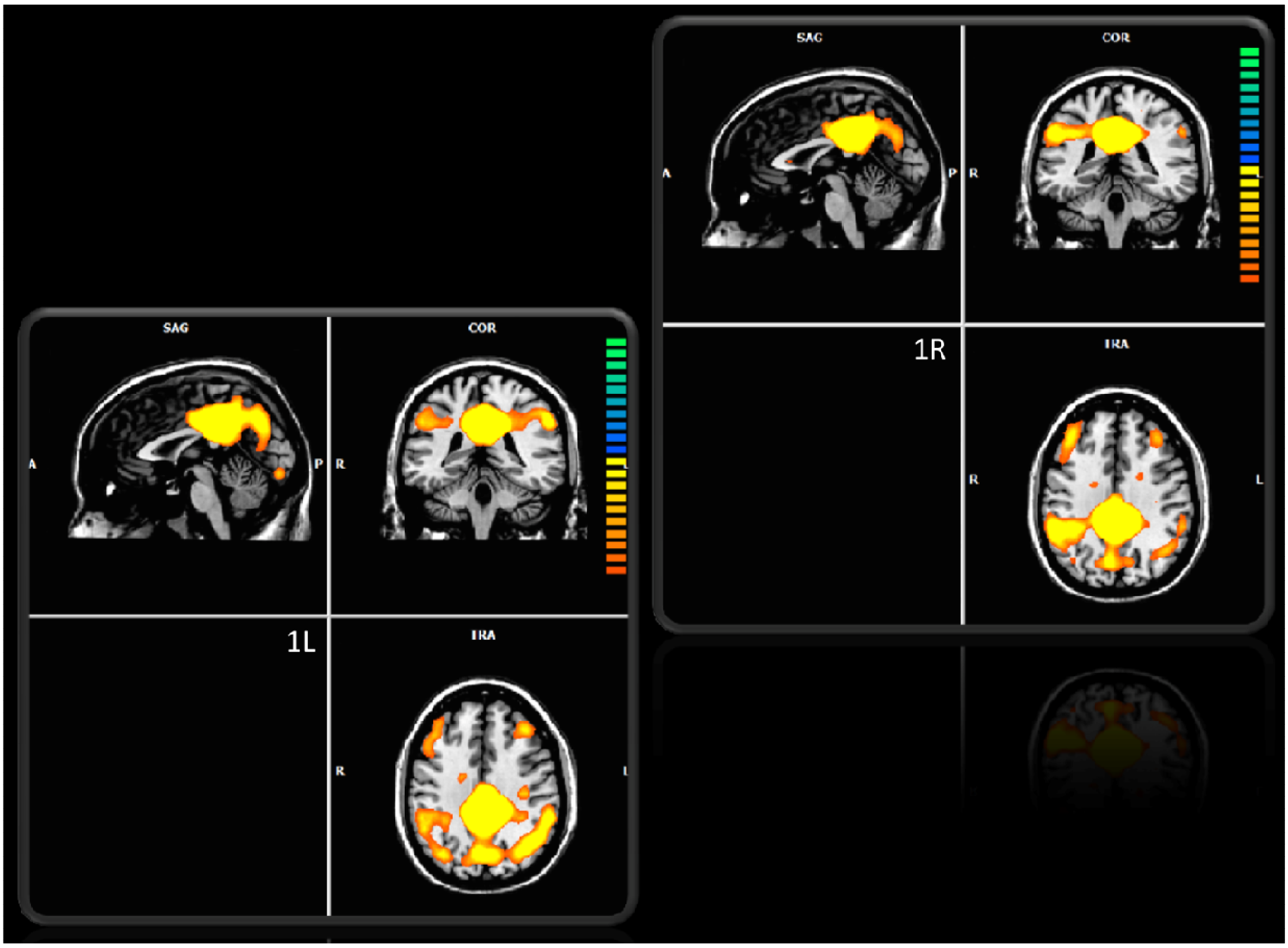

ROI 1 left (on the left) and right (on the right) rsFC

Fig S2. Effects of a displaced, increased or reduced ROI

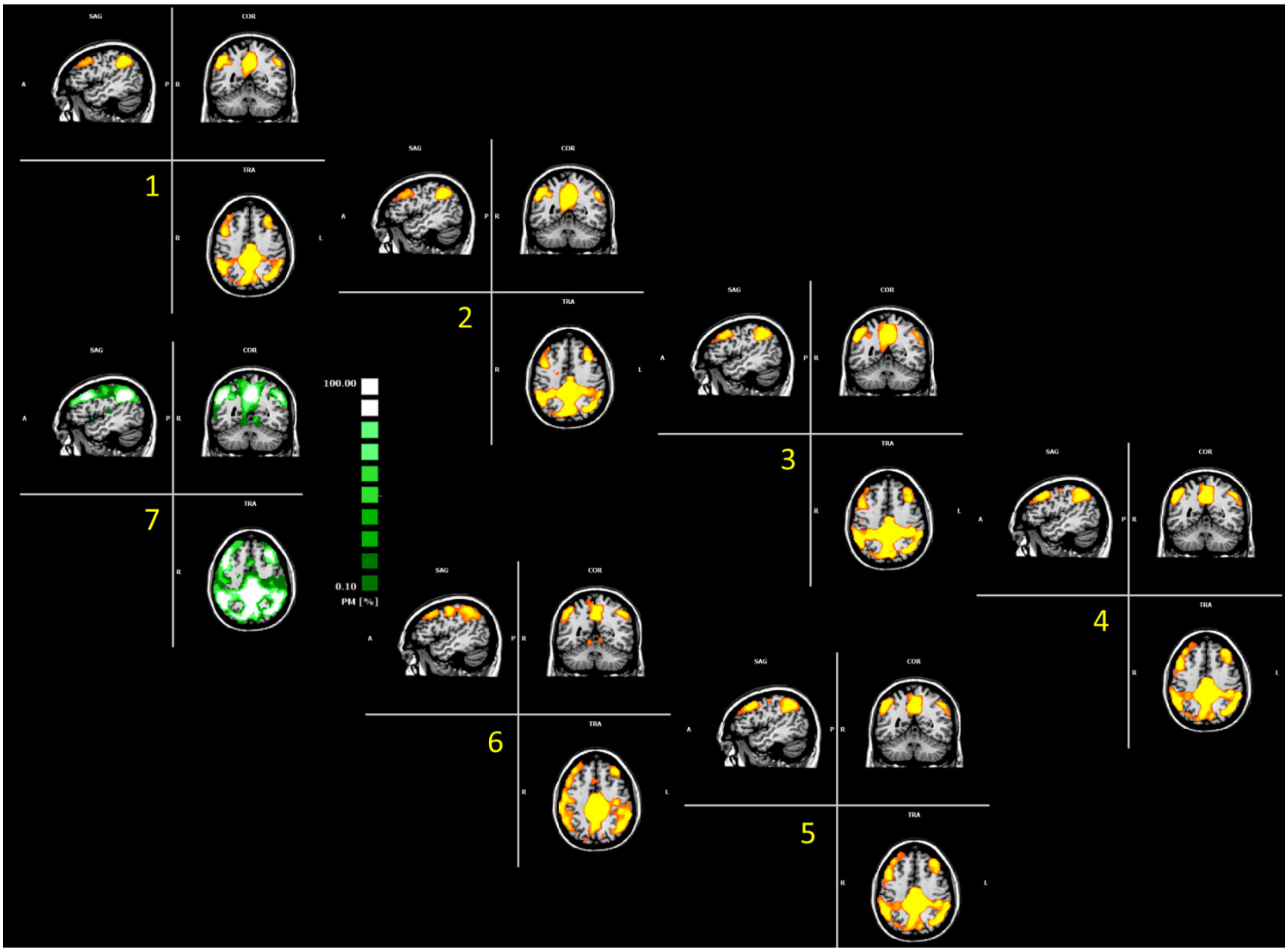

1-4 displacement: dorsal, rostral and caudal directions (3mm in each direction); 5 reduced dimension (27 mm^3); 6 increased dimension (512 mm^3); 7 Probabilistic map of all moved/increased/decreased ROI correlation pattern

Fig S3. Effect of the orthogonalization

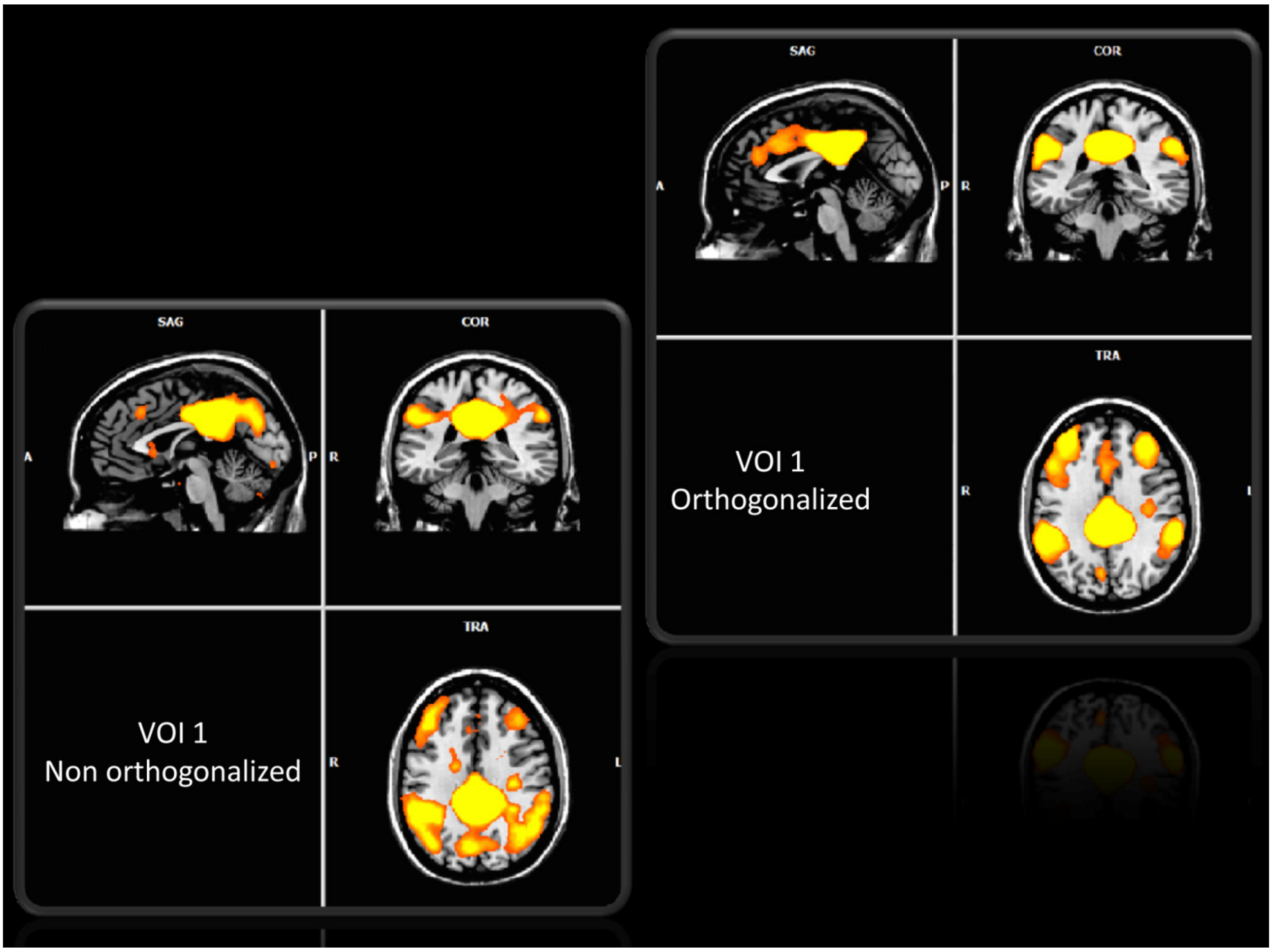

ortogonalized and non ortogonalized predictors (ROI1)

Fig S4. ROI 1 rsFC positive and negative correlations

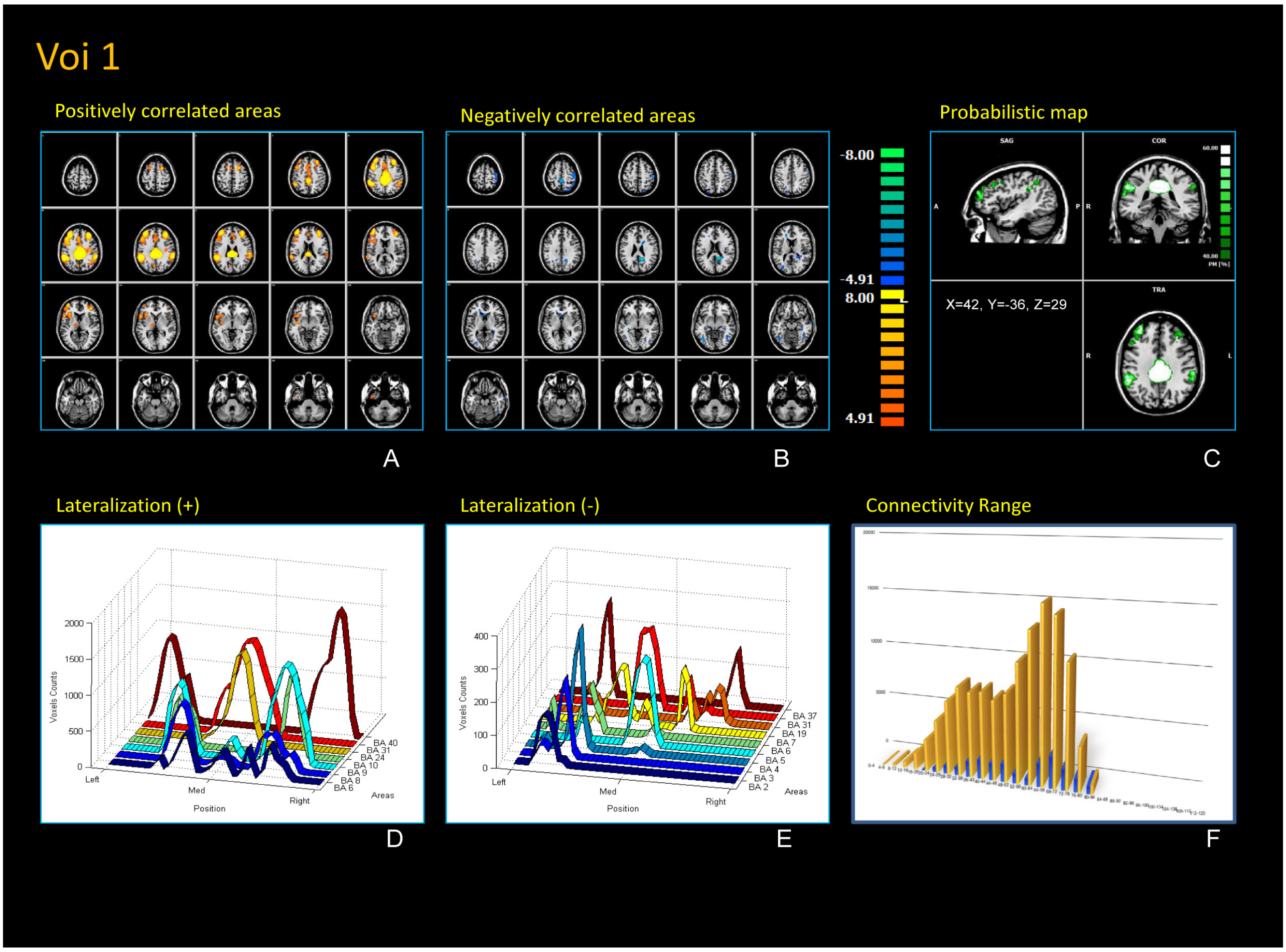

One sample t-test, corrections for multiple comparisons performed at the cluster level: q<0.05, cluster threshold K>16 voxels. Colors from red to yellow indicate positively correlated voxels. Colors from blue to green indicates negatively correlated voxels. Colors from green to white indicates negatively percentage of subjects overlapping. BA lateralization of the positively correlated areas. BA lateralization of the negatively correlated areas.
Connectivity range of positively (yellow) and negatively (blue) correlated voxels.

Fig S5. ROI 2 rsFC positive and negative correlations

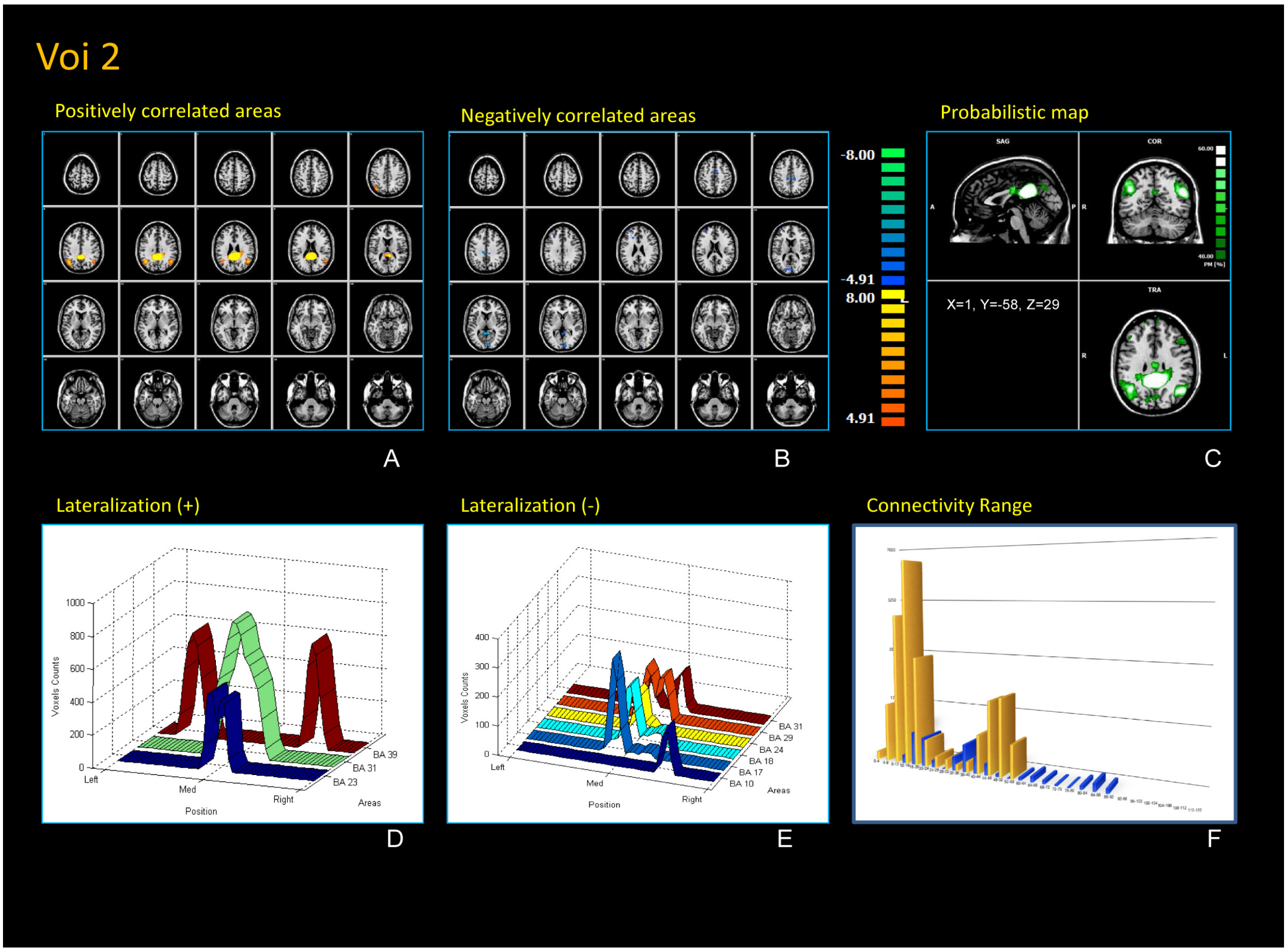

One sample t-test, corrections for multiple comparisons performed at the cluster level: q<0.05, cluster threshold K>16 voxels. Colors from red to yellow indicate positively correlated voxels. Colors from blue to green indicate negatively correlated voxels. Colors from green to white indicate negatively percentage of subjects overlapping. BA lateralization of the positively correlated areas. BA lateralization of the negatively correlated areas.
Connectivity range of positively (yellow) and negatively (blue) correlated voxels.

Fig S6. ROI 3 rsFC positive and negative correlations

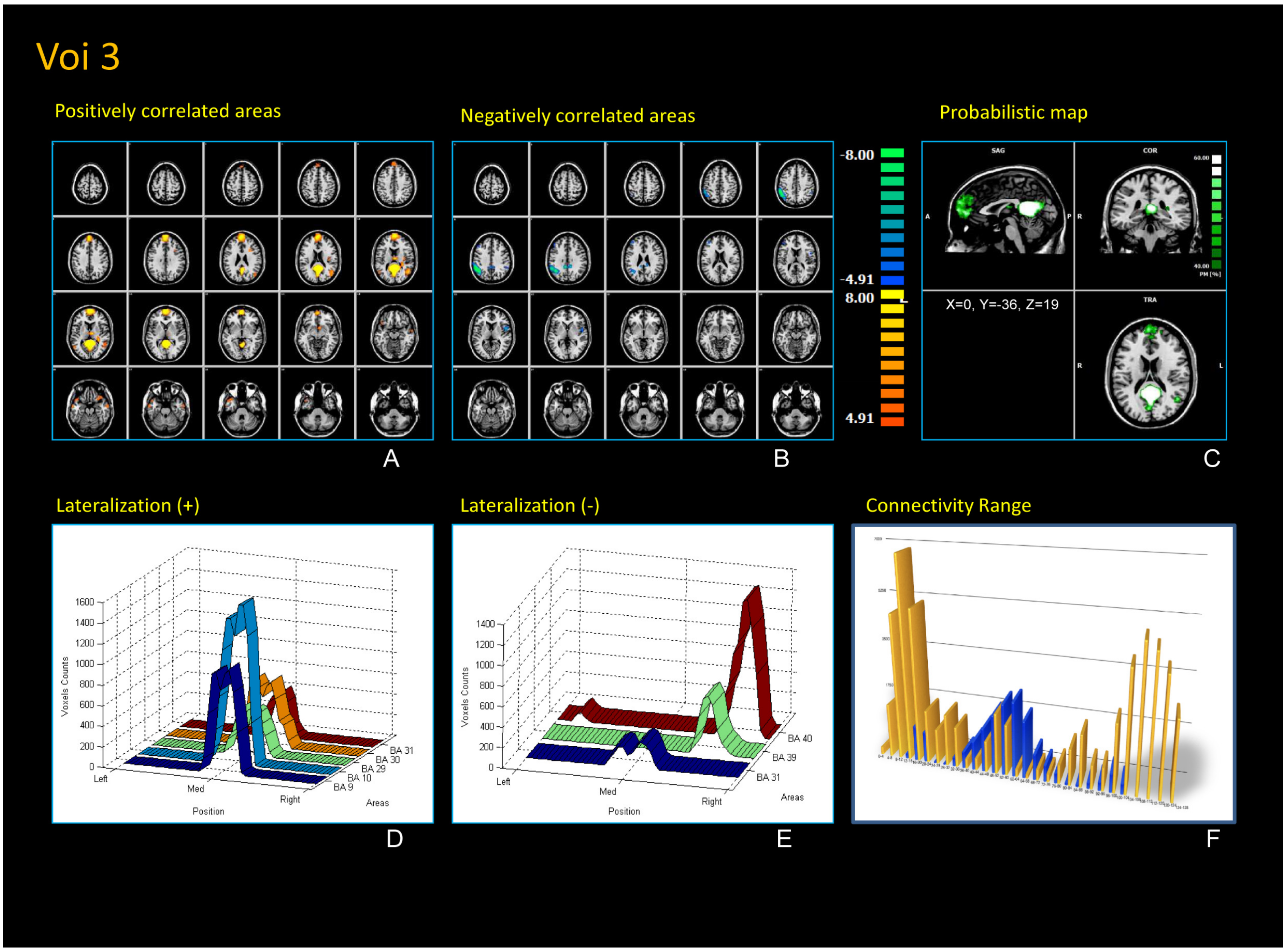

One sample t-test, corrections for multiple comparisons performed at the cluster level: q<0.05, cluster threshold K>16 voxels. Colors from red to yellow indicate positively correlated voxels. Colors from blue to green indicate negatively correlated voxels. Colors from green to white indicate negatively percentage of subjects overlapping. BA lateralization of the positively correlated areas. BA lateralization of the negatively correlated areas.
Connectivity range of positively (yellow) and negatively (blue) correlated voxels.

Fig S7. ROI 4 rsFC positive and negative correlations

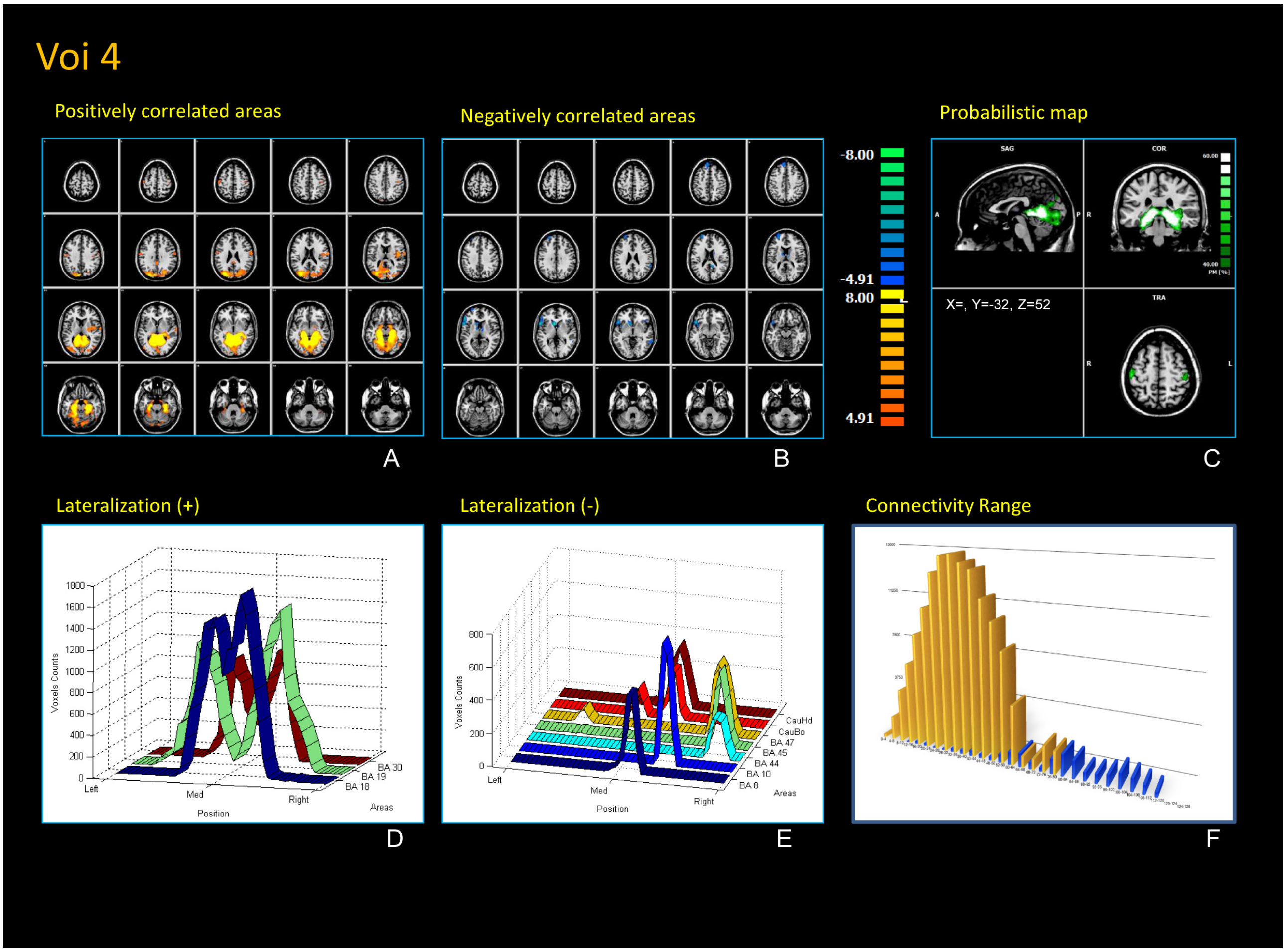

One sample t-test, corrections for multiple comparisons performed at the cluster level: q<0.05, cluster threshold K>16 voxels. Colors from red to yellow indicate positively correlated voxels. Colors from blue to green indicate negatively correlated voxels. Colors from green to white indicate negatively percentage of subjects overlapping. BA lateralization of the positively correlated areas. BA lateralization of the negatively correlated areas.
Connectivity range of positively (yellow) and negatively (blue) correlated voxels.

Fig S8. ROI 5 rsFC positive and negative correlations

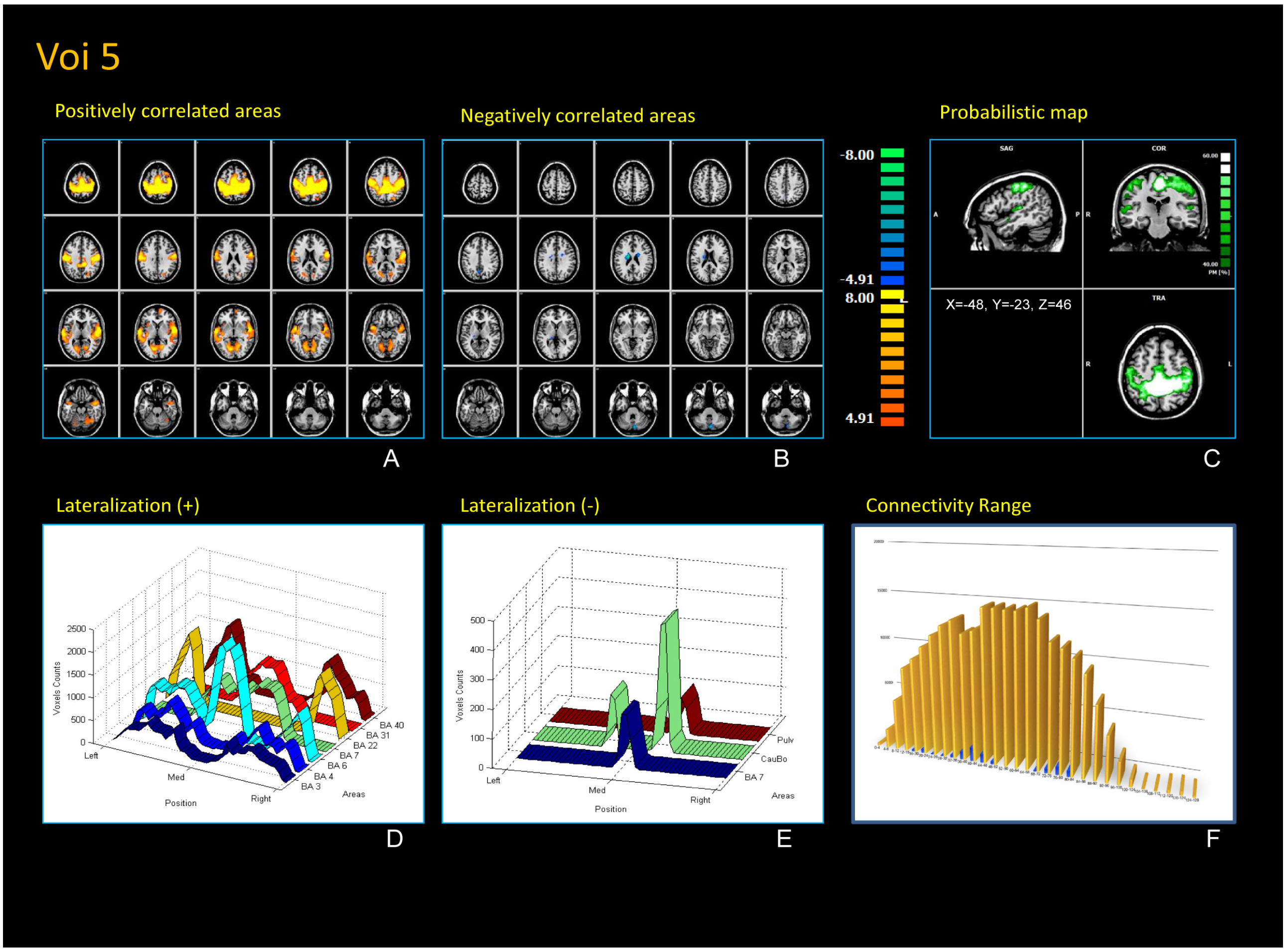

One sample t-test, corrections for multiple comparisons performed at the cluster level: q<0.05, cluster threshold K>16 voxels. Colors from red to yellow indicate positively correlated voxels. Colors from blue to green indicate negatively correlated voxels. Colors from green to white indicate negatively percentage of subjects overlapping. BA lateralization of the positively correlated areas. BA lateralization of the negatively correlated areas.
Connectivity range of positively (yellow) and negatively (blue) correlated voxels.

Fig S9. ROI 6 rsFC positive and negative correlations

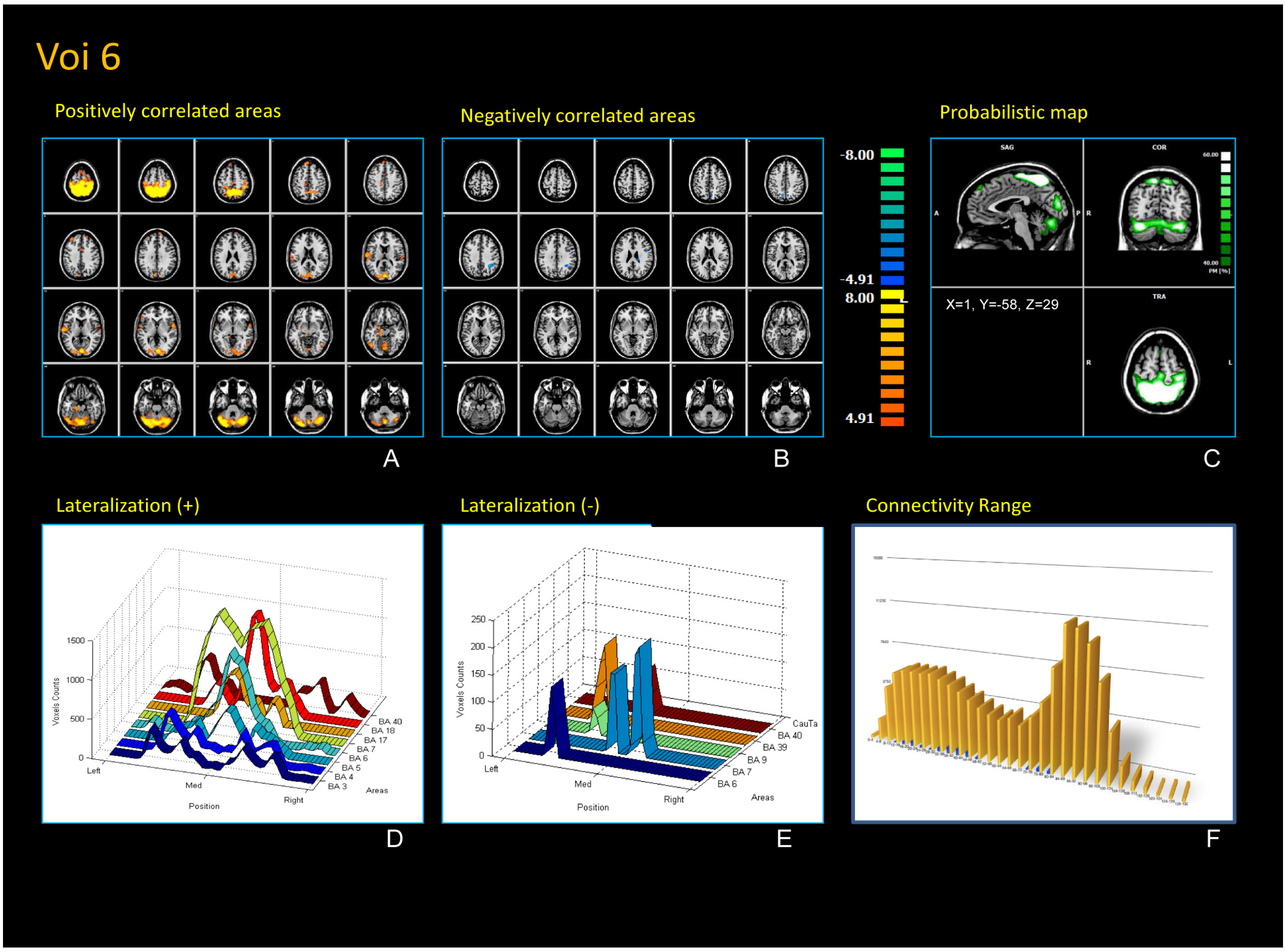

One sample t-test, corrections for multiple comparisons performed at the cluster level: q<0.05, cluster threshold K>16 voxels. Colors from red to yellow indicate positively correlated voxels. Colors from blue to green indicate negatively correlated voxels. Colors from green to white indicate negatively percentage of subjects overlapping. BA lateralization of the positively correlated areas. BA lateralization of the negatively correlated areas.
Connectivity range of positively (yellow) and negatively (blue) correlated voxels.

Fig S10. ROI 7 rsFC positive and negative correlations

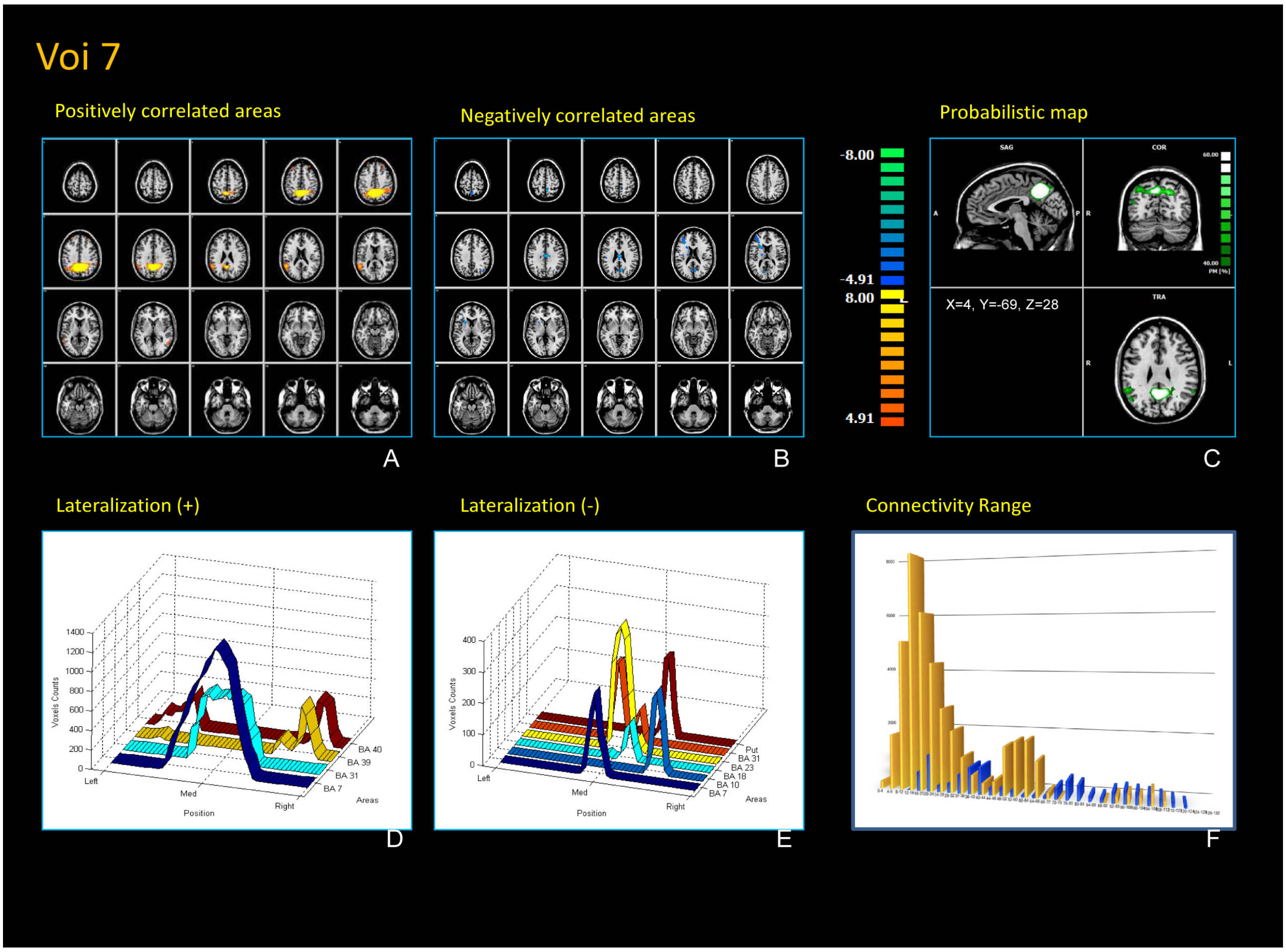

One sample t-test, corrections for multiple comparisons performed at the cluster level: q<0.05, cluster threshold K>16 voxels. Colors from red to yellow indicate positively correlated voxels. Colors from blue to green indicate negatively correlated voxels. Colors from green to white indicate negatively percentage of subjects overlapping. BA lateralization of the positively correlated areas. BA lateralization of the negatively correlated areas.
Connectivity range of positively (yellow) and negatively (blue) correlated voxels.

Fig S11. ROI 8 rsFC positive and negative correlations

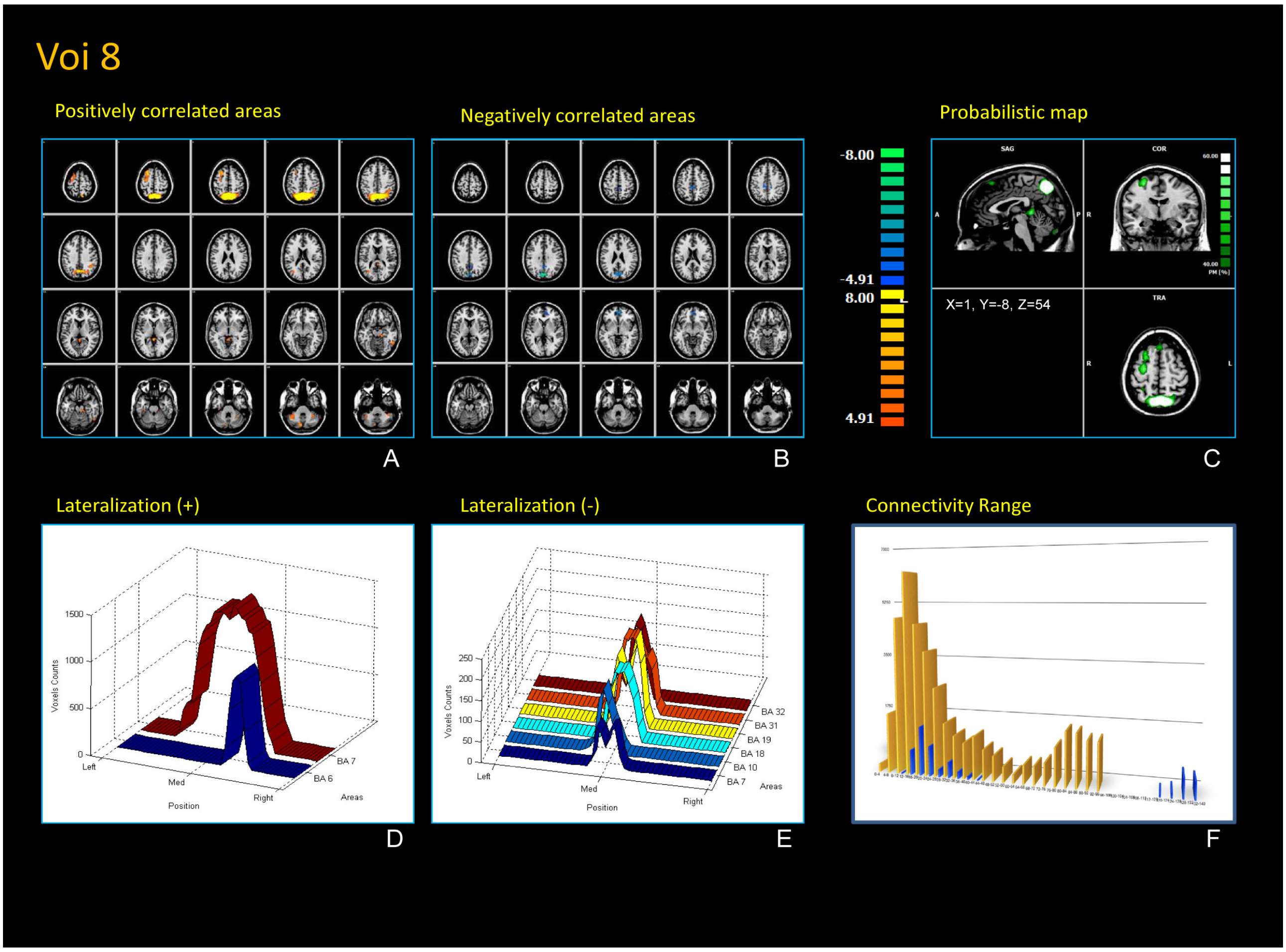

One sample t-test, corrections for multiple comparisons performed at the cluster level: q<0.05, cluster threshold K>16 voxels. Colors from red to yellow indicate positively correlated voxels. Colors from blue to green indicate negatively correlated voxels. Colors from green to white indicate negatively percentage of subjects overlapping. BA lateralization of the positively correlated areas. BA lateralization of the negatively correlated areas.
Connectivity range of positively (yellow) and negatively (blue) correlated voxels.

Fig S12. ROI 9 rsFC positive and negative correlations

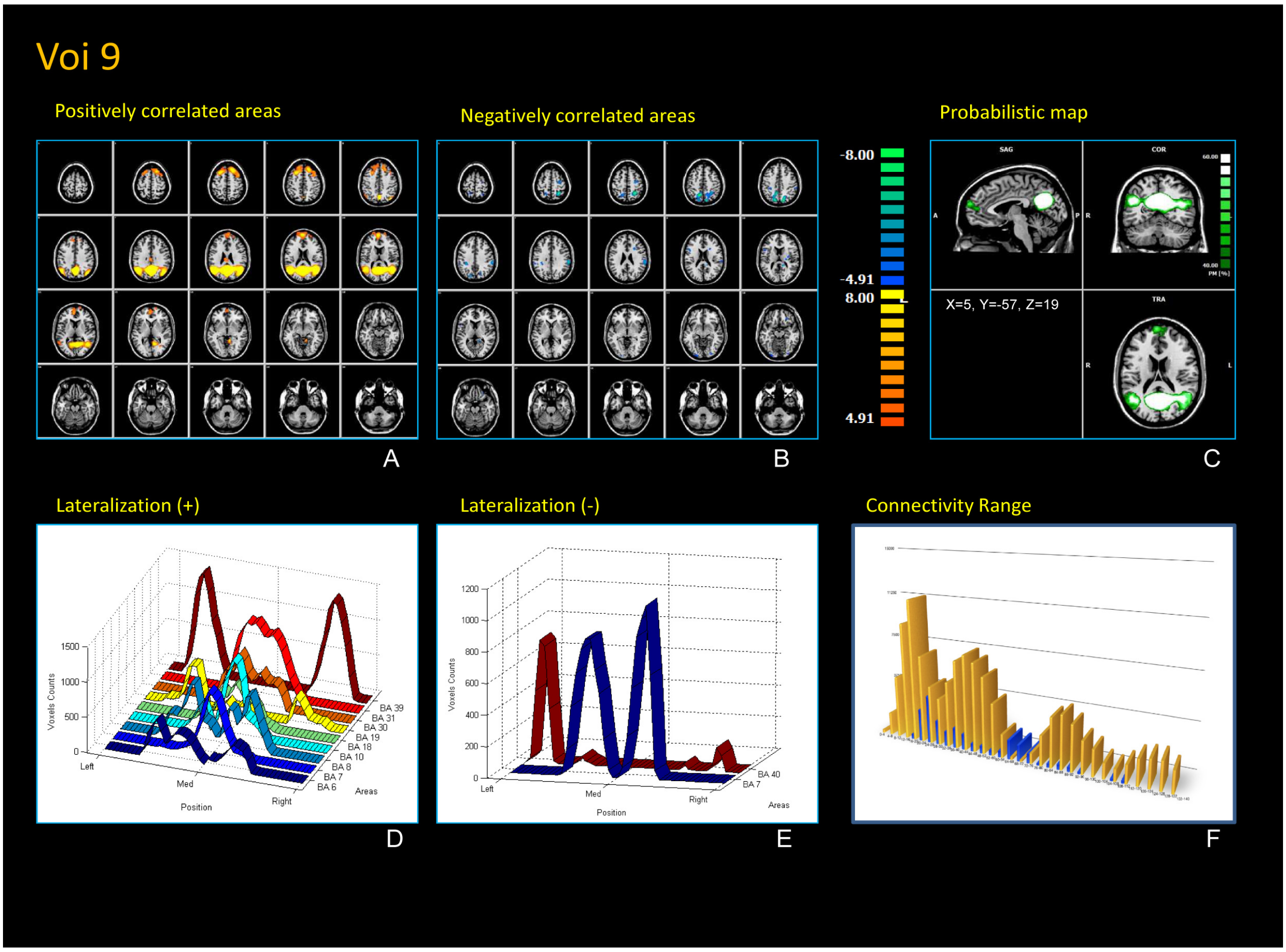

One sample t-test, corrections for multiple comparisons performed at the cluster level: q<0.05, cluster threshold K>16 voxels. Colors from red to yellow indicate positively correlated voxels. Colors from blue to green indicate negatively correlated voxels. Colors from green to white indicate negatively percentage of subjects overlapping. BA lateralization of the positively correlated areas. BA lateralization of the negatively correlated areas.
Connectivity range of positively (yellow) and negatively (blue) correlated voxels.

Fig S13. ROI 10 rsFC positive and negative correlations

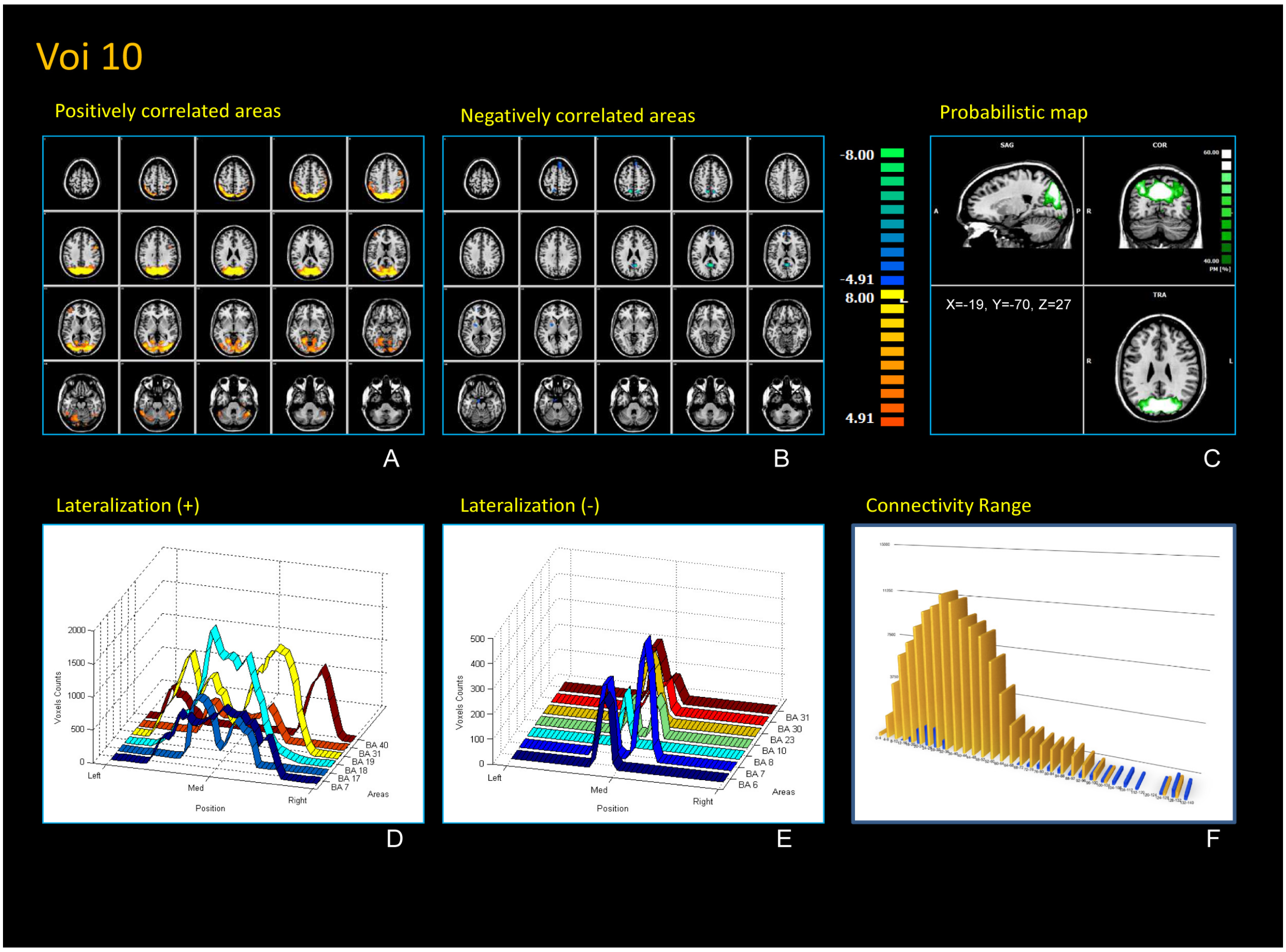

One sample t-test, corrections for multiple comparisons performed at the cluster level: q<0.05, cluster threshold K>16 voxels. Colors from red to yellow indicate positively correlated voxels. Colors from blue to green indicate negatively correlated voxels. Colors from green to white indicate negatively percentage of subjects overlapping. BA lateralization of the positively correlated areas. BA lateralization of the negatively correlated areas.
Connectivity range of positively (yellow) and negatively (blue) correlated voxels.

Fig S14. Conjunction analysis

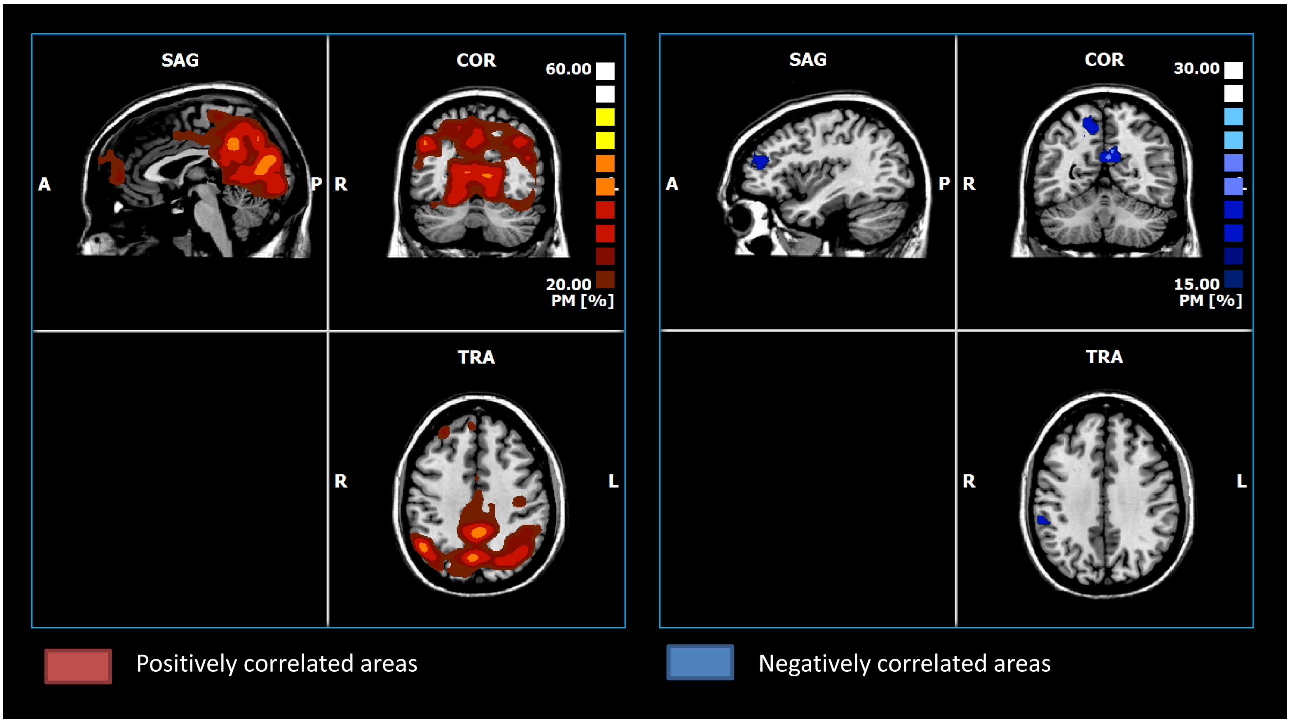

Conjunction of all positive (red) and negative (blue) correlations common to all ROIs.

Fig S15. ROI lateralization

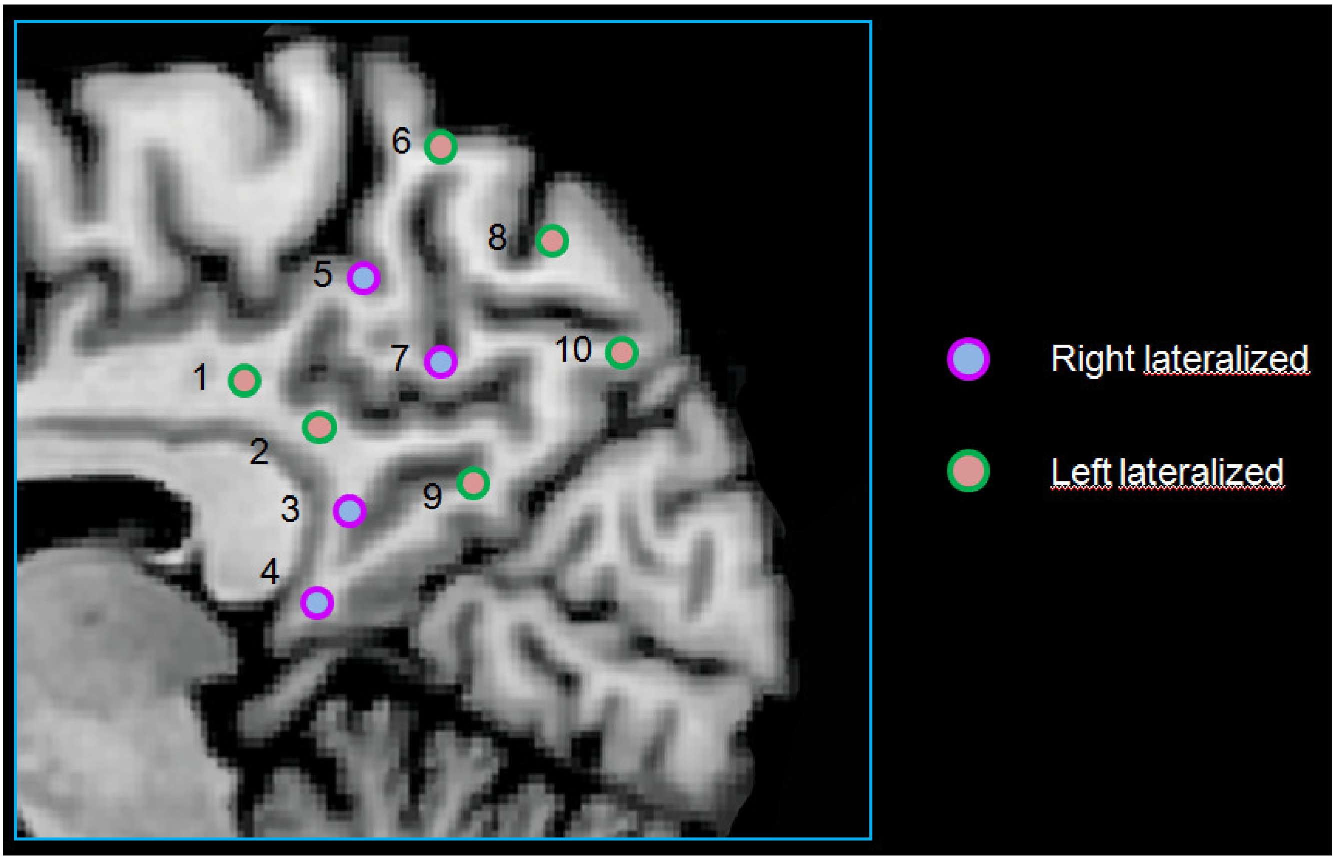

Prevalent lateralization of the ten designed ROI

Fig S16. Probabilistic maps of the four prevalent networks

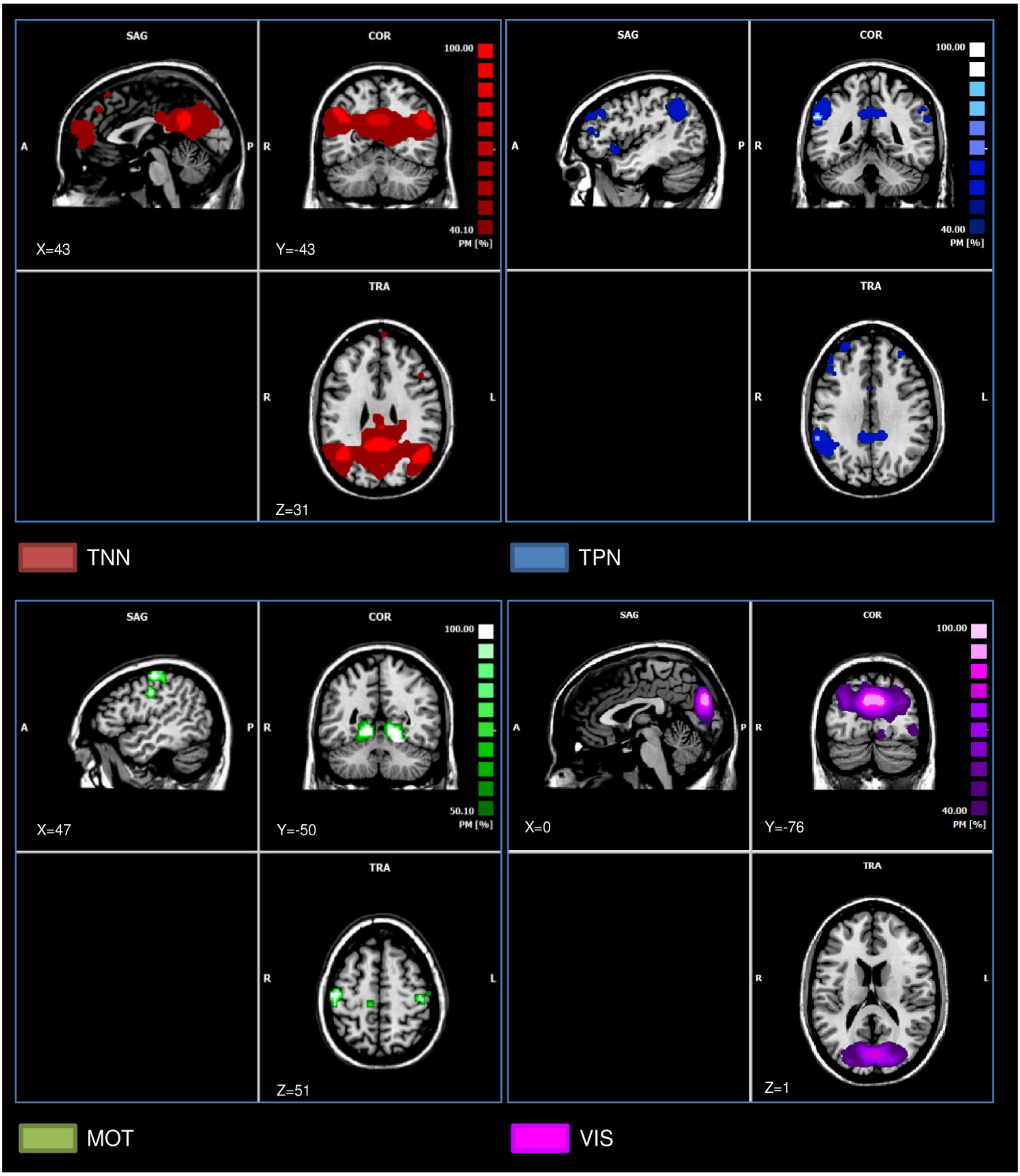

Probabilistic maps of the four prevalent networks.TNN: Task-Negative Network, TPN: Task-Positive Network , MOT: Sensorimotor Network, VIS: Visual Network.

Fig S17 Comparison with the results of the study by Margulies et al.

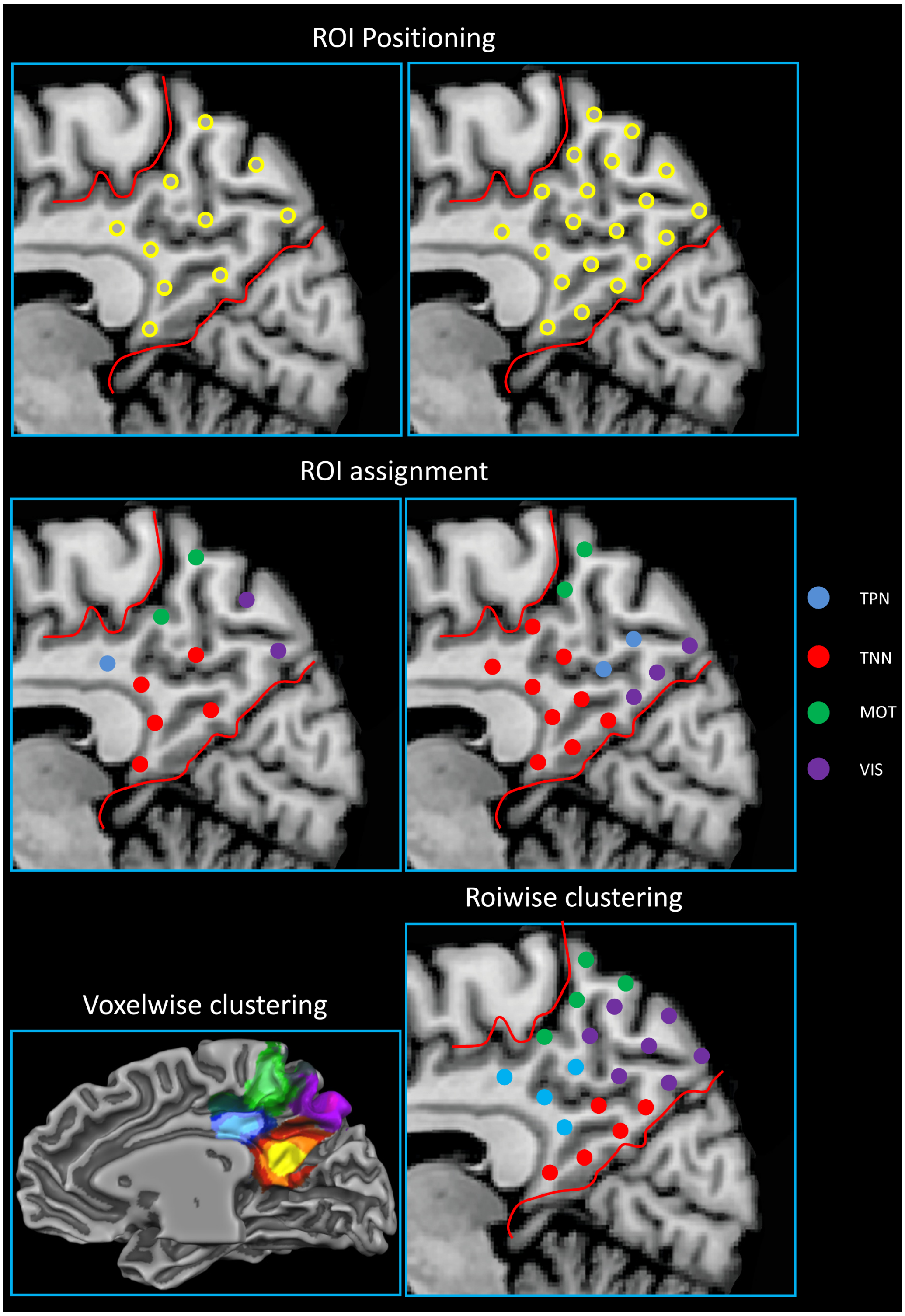

Left panels: data from the present study. Right panels: data from the study by Margulies et. al.

Fig S18 TPN connectivity pattern

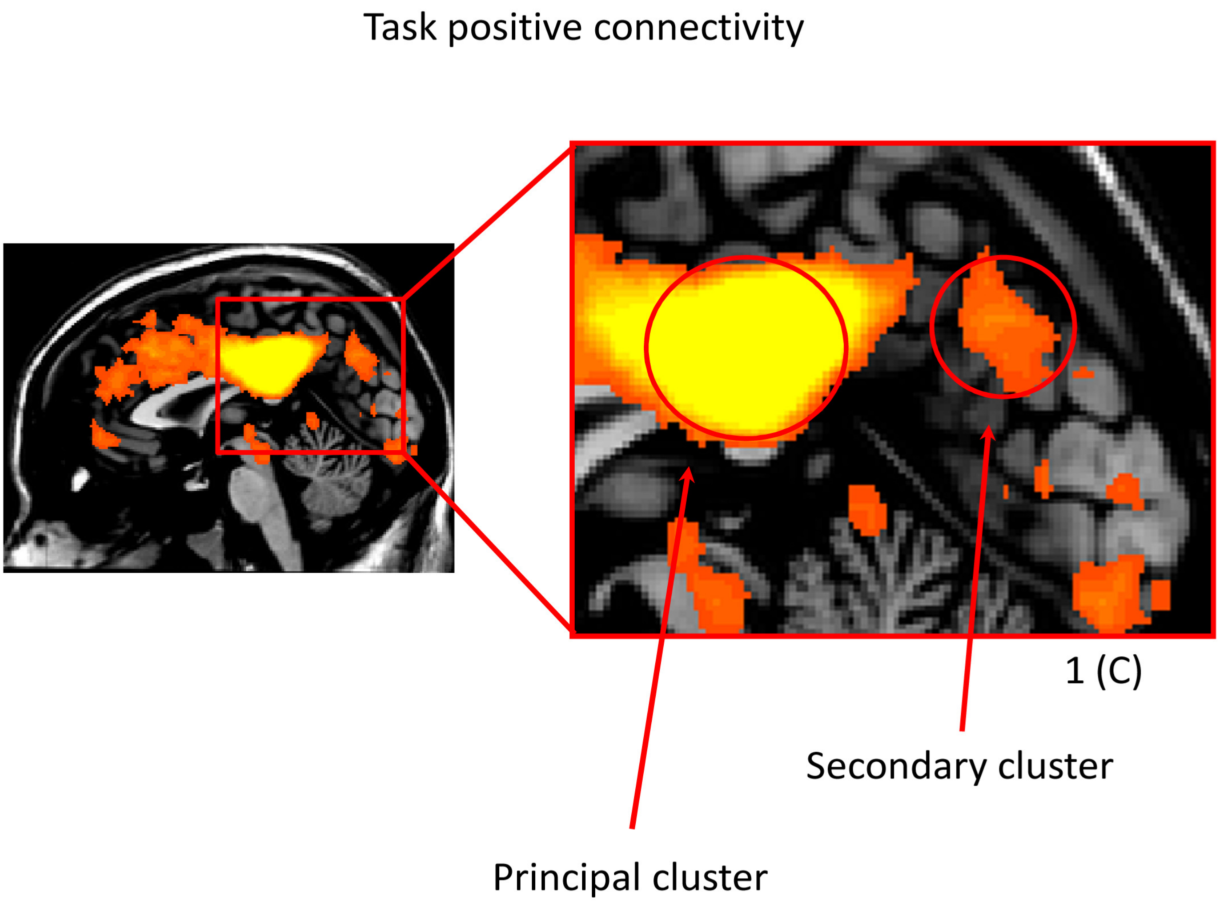

The TPN shows two blobs of connectivity in the posteromedial cortex

Supplementary Tables

Tab S1. Average coordinates of the ROI used as seed regions for rsFC analyses

ROI	X	Y	Z	Size (mm^3)
1	+-6	-32	32	125
2	+-6	-45	25	125
3	+-6	-46	14	125
4	+-8	-39	6	125
5	+-6	-43	42	125
6	+-6	-48	62	125
7	+-7	-50	36	125
8	+-6	-64	51	125
9	+-6	-57	29	125
10	+-6	-71	39	125

Tab S2. Subcortical correlations

BA	Positive	Negative
23	Thal DM (right)<br>Thal VL<br>Claustrum (right)	Hippocampus<br>Caudate (left)
29/30	Thal DM (left)<br>Caudate (left)<br>Claustrum (left)<br>Accumbens (left)<br>Amygdala	Thal DM<br>Caudate (right)
31	Claustrum (right)	Thal Pulvinar<br>Thal VL (right)<br>Putamen (right)<br>Claustrum<br>Caudate
7	Amygdala<br>Midbrain (left)?<br>Cerebellum	Putamen-Pallidum (right)

Thal = Thalamus, DM = Dorsal Medial, VL = Ventral Lateral

Tab S3. Correlations of each ROI with Brain Areas

AREA (BA) / ROI	1	2	3	4	5	6	7	8	9	10
MFG<br>(BA 9, 10)	X	O	X	O	X	X	O	O	X	O
ACC<br>(BA 24, 32, 33)	X	O						O	X	
DLPFC<br>(BA 44, 45, 46)	X			O						
Premotor cortex<br>(BA 6, 8)	X<br>O			O	X	X<br>O		X	X	O
MS cortex<br>(BA 4, 3, 2, 1)	O				X	X				
IPS<br>(BA 5, 7)	O				X<br>O	X<br>O	X<br>O	X<br>O	X<br>O	X<br>O
IPL/TPJ<br>(BA 39, 40)	X	X	O		X	X<br>O	X		X<br>O	X
Medial Temporal cortex<br>(BA 27, 28, 34, 35, 36)				X						
PCC<br>(BA 23, 26, 29, 30, 31)	X<br>O	X<br>O	X<br>O	X	X	X	X<br>O	O	X	X<br>O
Temporal neocortex<br>(BA 20, 21, 22, 37, 38)	O				X	X		X		
Visual cortex<br>(BA, 17, 18, 19)	O	O		X	X	X	O	O	X	X
Cerebellum					X	X		X		X

Positive (X) and negative (O) correlations. MFG = Middle Frontal Gyrus, ACC = Anterior Cingulate Cortex, DLPFC = Dorso Lateral Prefrontal Cortex, MS = Motor Sulcus, IPS = Intraparietal Sulcus, IPL = Inferior Parietal Lobule, TPJ = Temporo Parietal Junction, PCC = Posterior Cingulate Cortex

Tab S4. Correlations of each ROI with the TPN and the TNN

ROI	TPN							TNN						
	SPL 7	IPL 40	FEF 8	SMA 6	DL PFC	MT 19 37	Ins	PCC 31	rSPL 30	TPJ 39	Me PFC 10 32	SF 8	IT 20 21	PHG 35
**1**	O	X	X		X		X	X<br>O			X	X		
**2**								X<br>O		X	O			
**3**		O					X	X<br>O	X	O	X		X	
**4**					O	X	X		X		O	O		X
**5**	X<br>O	X	X	X			X	X			X*			
**6**	X<br>O	X<br>O				X				O*				
**7**	X<br>O	X						X<br>O		X	O			
**8**	X<br>O		X					O			O			
**9**	X<br>O	O						X	X	X	X	X*		
**10**	X<br>O	X	X			X		X<br>O	O		O	O		

Positive (X) and negative (O) correlations with the task-positive network (TPN) and the task-negative network (TNN). SPL = Superior Parietal Lobule, FEF = Frontal Eys Field, MT = Middle Temporal, Ins = Insula, rSPL = retro Splenial cortex, MePFC = Medial Prefrontal Cortex, SF = Superior Frontal, IT = Inferior Temporal, PHG = Parahippocampal Gyrus. Other abbreviation as in Tab S3. * Only in the left hemisphere

Tab S5. Spearman Brown split half reliability index

ROI	RSB
1	0,62
2	0,62
3	0,64
4	0,62
5	0,70
6	0,61
7	0,70
8	0,75
9	0,72
10	0,72
Mean	0.67
Standard Deviation	0.05

Calculated for each ROI of the two split groups

SUPPLEMENTARY REFERENCES

1. Kriegeskorte N, Goebel R (2001) An efficient algorithm for topologically correct segmentation of the cortical sheet in anatomical mr volumes. Neuroimage 14: 329-346.
2. Goebel R (2000) A fast automated method for flattening cortical surfaces. Neuroimage 11: S680.
3. Goebel R, Hasson U, Lefi I, Malach R (2004) Statistical analyses across aligned cortical hemispheres reveal high-resolution population maps of human visual cortex. Neuroimage 22.
4. van Atteveldt N, Formisano E, Goebel R, Blomert L (2004) Integration of letters and speech sounds in the human brain. Neuron 43: 271-282.
5. Forman SD, Cohen JD, Fitzgerald M, Eddy WF, Mintun MA, et al. (1995) Improved assessment of significant activation in functional magnetic resonance imaging (fMRI): use of a cluster-size threshold. Magn Reson Med 33: 636-647.
6. Goebel R, Esposito F, Formisano E (2006) Analysis of functional image analysis contest (FIAC) data with brainvoyager QX: From single-subject to cortically aligned group general linear model analysis and self-organizing group independent component analysis. Hum Brain Mapp 27: 392-401.
7. Zadeh LA (1977) Fuzzy Set and Their Application to Pattern Recognition and Clustering Analysis. In: Van Ryzin J, editor. Classification and clustering : proceedings of an Advanced Seminar conducted by the Mathematics Research Center, the University of Wisconsin at Madison, May 3-5, 1976. New York ; London: Academic Press. pp. 355-393.
8. Bezdek JC, Ehrlich R, Full W (1984) FCM: The fuzzy c-means clustering algorithm. Computers & Geosciences 10: 191-203.
9. Mahalanobis PC (1936) On the generalised distance in statistics. Proc Natl Acad Sci U S A 2.
10. Fadili MJ, Ruan S, Bloyet D, Mazoyer B (2001) On the number of clusters and the fuzziness index for unsupervised FCA application to BOLD fMRI time series. Med Image Anal 5: 55-67.
11. Wang W, Zhang Y (2007) On fuzzy cluster validity indices. Fuzzy Sets Sys 158: 2095-2117.
12. Esposito F, Scarabino T, Hyvarinen A, Himberg J, Formisano E, et al. (2005) Independent component analysis of fMRI group studies by self-organizing clustering. Neuroimage 25: 193-205.
13. Himberg J, Hyvarinen A, Esposito F (2004) Validating the independent components of neuroimaging time series via clustering and visualization. Neuroimage 22: 1214-1222.

14. Fair DA, Cohen AL, Dosenbach NU, Church JA, Miezin FM, et al. (2008) The maturing architecture of the brain's default network. Proc Natl Acad Sci U S A 105: 4028-4032.

15. Stevens MC, Pearlson GD, Calhoun VD (2009) Changes in the interaction of resting-state neural networks from adolescence to adulthood. Hum Brain Mapp.

16. Greicius MD, Krasnow B, Reiss AL, Menon V (2003) Functional connectivity in the resting brain: a network analysis of the default mode hypothesis. Proc Natl Acad Sci U S A 100: 253-258.

17. Raichle ME, MacLeod AM, Snyder AZ, Powers WJ, Gusnard DA, et al. (2001) A default mode of brain function. Proc Natl Acad Sci U S A 98: 676-682.

18. Raichle ME, Snyder AZ (2007) A default mode of brain function: a brief history of an evolving idea. Neuroimage 37: 1083-1090; discussion 1097-1089.

19. Fox MD, Snyder AZ, Vincent JL, Corbetta M, Van Essen DC, et al. (2005) The human brain is intrinsically organized into dynamic, anticorrelated functional networks. Proc Natl Acad Sci U S A 102: 9673-9678.

20. De Luca M, Beckmann CF, De Stefano N, Matthews PM, Smith SM (2006) fMRI resting state networks define distinct modes of long-distance interactions in the human brain. Neuroimage 29: 1359-1367.

21. Fransson P (2006) How default is the default mode of brain function? Further evidence from intrinsic BOLD signal fluctuations. Neuropsychologia 44: 2836-2845.

22. Shehzad Z, Kelly AM, Reiss PT, Gee DG, Gotimer K, et al. (2009) The Resting Brain: Unconstrained yet Reliable. Cereb Cortex.

23. Wu CW, Gu H, Lu H, Stein EA, Chen JH, et al. (2009) Mapping functional connectivity based on synchronized CMRO2 fluctuations during the resting state. Neuroimage 45: 694-701.

24. Damoiseaux JS, Rombouts SA, Barkhof F, Scheltens P, Stam CJ, et al. (2006) Consistent resting-state networks across healthy subjects. Proc Natl Acad Sci U S A 103: 13848-13853.

25. Luo L, O'Leary DD (2005) Axon retraction and degeneration in development and disease. Annu Rev Neurosci 28: 127-156.

26. Fox MD, Raichle ME (2007) Spontaneous fluctuations in brain activity observed with functional magnetic resonance imaging. Nat Rev Neurosci 8: 700-711.

27. Margulies DS, Vincent JL, Kelly C, Lohmann G, Uddin LQ, et al. (2009) Precuneus shares intrinsic functional architecture in humans and monkeys. Proc Natl Acad Sci U S A 106: 20069-20074.

28. Goebel R, Roebroeck A, Kim DS, Formisano E (2003) Investigating directed cortical interactions in time-resolved fMRI data using vector autoregressive modeling and Granger causality mapping. Magn Reson Imaging 21: 1251-1261.

29. Roebroeck A, Formisano E, Goebel R (2005) Mapping directed influence over the brain using Granger causality and fMRI. Neuroimage 25: 230-242.
